# Supplementary material for: First Report of Two Gymnodimines and Two Tetrodotoxin Analogues in Invertebrates from the North Atlantic Coast of Spain
Source: Mar Drugs. 2023 Apr 5;21(4):232. doi: 10.3390/md21040232 (PMC10144553; doi:10.3390/md21040232)
Supplement: Supplementary file 1 [file marinedrugs-21-00232-s001.zip › marinedrugs-2253671-supplementary.pdf]

*Supplementary material*

# First report of two gymnodimines and two tetrodotoxin analogues in invertebrates from the north Atlantic Coast of Spain

Table S1. Date, sampling location, Ría, class, species and type of analyzed toxins performed in the study between May 2021 to October 2022. Samples with the analyzed toxins marked in red were positive for that compound.

| Date | Sampling location | Ria | Class | Species | Analyzed toxins |
|---|---|---|---|---|---|
| May 11, 2021 | Ferrol. As Pías | Ferrol | Bivalve | *Modiolus modiolus* | TTX |
| May 11, 2021 | Ferrol. As Pías | Ferrol | Bivalve | *Acanthocardia tuberculata* | TTX |
| May 25, 2021 | Vilanova de Arousa. Corón | Arousa | Bivalve | *Ensis siliqua* | TTX |
| May 25, 2021 | Vilanova de Arousa. Corón | Arousa | Bivalve | *Ruditapes philippinarum* | TTX |
| May 25, 2021 | Vilanova de Arousa. Corón | Arousa | Bivalve | *Cerastoderma edule* | TTX |
| May 25, 2021 | Vilanova de Arousa. Corón | Arousa | Bivalve | *Mytilus galloprovincialis* | TTX |
| May 25, 2021 | Vilanova de Arousa. Corón | Arousa | Crustacean | *Polybius sp.* | TTX |
| May 25, 2021 | Vilanova de Arousa. Corón | Arousa | Crustacean | *Polybius sp.* | TTX |
| May 25, 2021 | Vilanova de Arousa. Corón | Arousa | Gastropod | *Ocenebra sp.* | TTX |
| May 25, 2021 | Lira. Ximpron | Corcubión | Echinoderm | *n.i.* | TTX |
| May 26, 2021 | MurosI. Esteiro | Muros-Noia | Gastropod | *Patella sp.* | TTX |
| June 29, 2021 | Barallobre | Ferrol | Bivalve | *Mytilus galloprovincialis* | TTX |
| June 29, 2021 | Miño | Betanzos | Bivalve | *Ruditapes philippinarum* | TTX |
| June 29, 2021 | Pasaxe. Sta Cristina | A Coruña | Bivalve | *Cerastoderma edule* | TTX |
| June 29, 2021 | Camariñas | Camariñas | Bivalve | *Mytilus galloprovincialis* | TTX |
| June 30, 2021 | Corcubión. Cee | Corcubión | Bivalve | *Mytilus galloprovincialis* | TTX |

| | | | | | |
|---|---|---|---|---|---|
| June 30, 2021 | Barqueiro | Barqueiro | Bivalve | *Cerastoderma edule* | TTX |
| July 2, 2021 | Arousa I. Meloxo | Arousa | Bivalve | *Venerupis pullastra* | TTX |
| July 2, 2021 | Arousa VI | Arousa | Bivalve | *Venerupis pullastra* | TTX |
| July 2, 2021 | Muros I. Abelleira | Muros-Noia | Bivalve | *Cerastoderma edule* | TTX |
| July 5, 2021 | Muros I | Muros-Noia | Bivalve | *Cerastoderma edule* | TTX |
| July 6, 2021 | Barqueiro. San Fiz | Barqueiro | Bivalve | *Magellana gigas* | TTX |
| July 6, 2021 | Miño. Lombo Espiñeira | Betanzos | Bivalve | *Ruditapes philippinarum* | TTX |
| July 6, 2021 | Pasaxe. Sta Cristina | A Coruña | Bivalve | *Cerastoderma edule* | TTX |
| July 6, 2021 | Camariñas. Enseada Vasa | Camariñas | Bivalve | *Mytilus galloprovincialis* | TTX |
| July 6, 2021 | Arousa VI. Sarrido | Arousa | Bivalve | *Ruditapes philippinarum* | TTX |
| July 7, 2021 | Corcubión. Cee | Corcubión | Bivalve | *Cerastoderma edule* | TTX |
| July 7, 2021 | Arousa I. Meloxo | Arousa | Bivalve | *Venerupis pullastra* | TTX |
| July 8, 2021 | Barallobre. Rampa | Ferrol | Bivalve | *Mytilus galloprovincialis* | TTX |
| July 13, 2021 | Barallobre. Rampa | Ferrol | Bivalve | *Mytilus galloprovincialis* | TTX |
| July 13, 2021 | Miño. Lombo Espiñeira | Betanzos | Bivalve | *Ruditapes philippinarum* | TTX |
| July 13, 2021 | Pasaxe. Sta Cristina | A Coruña | Bivalve | *Cerastoderma edule* | TTX |
| July 13, 2021 | Camariñas. Enseada Vasa | Camariñas | Bivalve | *Mytilus galloprovincialis* | TTX |
| July 13, 2021 | Corcubión. Cee | Corcubión | Bivalve | *Mytilus galloprovincialis* | TTX |
| July 14, 2021 | Barqueiro. Salgueira | Barqueiro | Bivalve | *Mytilus galloprovincialis* | TTX |
| July 15, 2021 | Camariñas. Rio da Ponte | Camariñas | Bivalve | *Ruditapes decussatus* | TTX |
| July 20, 2021 | Barqueiro. Salgueira | Barqueiro | Bivalve | *Mytilus galloprovincialis* | TTX |
| July 20, 2021 | Pasaxe. Sta Cristina | A Coruña | Bivalve | *Cerastoderma edule* | TTX |
| July 21, 2021 | Miño. Lombo Espiñeira | Betanzos | Bivalve | *Ruditapes philippinarum* | TTX |
| July 21, 2021 | Camariñas. Rio da Ponte | Camariñas | Bivalve | *Ruditapes decussatus* | TTX |
| July 21, 2021 | Corcubión. Cee | Corcubión | Bivalve | *Mytilus galloprovincialis* | TTX |
| July 23, 2021 | Muros I. Abelleira | Muros-Noia | Bivalve | *Cerastoderma edule* | TTX |
| July 26, 2021 | Vilanova de Arousa. Corón | Arousa | Cnidaria | *n.i.* | TTX |
| July 26, 2021 | Vilanova de Arousa. Corón | Arousa | Gastropod | *n.i.* | TTX |
| July 26, 2021 | Vilanova de Arousa. Corón | Arousa | Gastropod | *Gibbula sp.* | TTX |
| July 26, 2021 | Vilanova de Arousa. Corón | Arousa | Bivalve | *Mytilus galloprovincialis* | TTX |
| July 26, 2021 | Vilanova de Arousa. Corón | Arousa | Gastropod | *Nucella sp.* | TTX |

| | | | | | |
|---|---|---|---|---|---|
| July 26, 2021 | Vilanova de Arousa. Corón | Arousa | Gastropod | *Littorina sp.* | TTX |
| July 26, 2021 | Vilanova de Arousa. Corón | Arousa | Gastropod | *Patella sp.* | TTX |
| July 27, 2021 | Barqueiro. Salgueira | Barqueiro | Bivalve | *Mytilus galloprovincialis* | TTX |
| July 27, 2021 | Miño. Lombo Espiñeira | Betanzos | Bivalve | *Ruditapes philippinarum* | TTX |
| July 27, 2021 | Pasaxe. Sta Cristina | A Coruña | Bivalve | *Cerastoderma edule* | TTX |
| July 27, 2021 | Camariñas | Camariñas | Bivalve | *Ruditapes decussatus* | TTX |
| July 27, 2021 | Muros I. Abelleira | Muros-Noia | Bivalve | *Cerastoderma edule* | TTX |
| July 27, 2021 | Muros III. Testal | Muros-Noia | Bivalve | *Cerastoderma edule* | TTX |
| July 28, 2021 | Pont V. Placeres | Pontevedra | Bivalve | *Ruditapes philippinarum* | TTX |
| July 29, 2021 | Corcubión. Cee | Corcubión | Bivalve | *Mytilus galloprovincialis* | TTX |
| July 29, 2021 | Pont II. Niño do Corvo | Pontevedra | Bivalve | *Ensis ensis* | TTX |
| July 29, 2021 | Vigo I. Cies | Vigo | Bivalve | *Venerupis rhomboides* | TTX |
| July 29, 2021 | Vigo II.2 Xunqueira | Vigo | Bivalve | *Ruditapes philippinarum* | TTX |
| August 3, 2021 | Barqueiro. San Fiz | Barqueiro | Bivalve | *Mytilus galloprovincialis* | TTX |
| August 3, 2021 | Corcubión. Cee | Corcubión | Bivalve | *Mytilus galloprovincialis* | TTX |
| August 3, 2021 | Pont V. Campelo | Pontevedra | Bivalve | *Ruditapes philippinarum* | TTX |
| August 3, 2021 | Vigo II.2 Xunqueira | Vigo | Bivalve | *Ruditapes philippinarum* | TTX |
| August 4, 2021 | Miño. Lombo Espiñeira | Betanzos | Bivalve | *Venerupis pullastra* | TTX |
| August 4, 2021 | Vigo I. Cies | Vigo | Bivalve | *Venerupis rhomboides* | TTX |
| August 5, 2021 | Rianxo. Porrón | Arousa | Gastropod | *Littorina sp.* | TTX |
| August 5, 2021 | Rianxo. Porrón | Arousa | Crustacean | *Polybius sp.* | TTX |
| August 5, 2021 | Rianxo. Porrón | Arousa | Bivalve | *Mytilus galloprovincialis* | TTX |
| August 5, 2021 | Pont II. San Cibrao | Pontevedra | Bivalve | *Ruditapes philippinarum* | TTX |
| August 6, 2021 | Vilanova de Arousa. Corón | Arousa | Gastropod | *Nucella sp.* | TTX |
| August 6, 2021 | Vilanova de Arousa. Corón | Arousa | Bivalve | *Ruditapes philippinarum* | TTX |
| August 6, 2021 | Vilanova de Arousa. Corón | Arousa | Bivalve | *Cerastoderma edule* | TTX |
| August 6, 2021 | Vilanova de Arousa. Corón | Arousa | Bivalve | *Mytilus galloprovincialis* | TTX |
| August 6, 2021 | Vilanova de Arousa. Corón | Arousa | Gastropod | *Littorina sp.* | TTX |
| August 6, 2021 | Vilanova de Arousa. Corón | Arousa | Gastropod | *Littorina sp.* | TTX |
| August 6, 2021 | Vilanova de Arousa. Corón | Arousa | Cnidaria | *n.i.* | TTX |
| August 6, 2021 | Vilanova de Arousa. Corón | Arousa | Gastropod | *n.i.* | TTX |

| | | | | | |
|---|---|---|---|---|---|
| August 6, 2021 | Vilanova de Arousa. Corón | Arousa | Crustacean | *Balanus sp.* | TTX |
| August 6, 2021 | Vilanova de Arousa. Corón | Arousa | Gastropod | *Patella sp.* | TTX |
| August 6, 2021 | Barallobre. Rampa | Ferrol | Bivalve | *Mytilus galloprovincialis* | TTX |
| August 6, 2021 | Camariñas. Enseada Vasa | Camariñas | Bivalve | *Ruditapes decussatus* | TTX |
| August 9, 2021 | Nerga. Os Castros | Vigo | Gastropod | *Patella sp.* | TTX |
| August 9, 2021 | Nerga. Os Castros | Vigo | Echinoderm | *n.i.* | TTX |
| August 9, 2021 | Nerga. Os Castros | Vigo | Cnidaria | *n.i.* | TTX |
| August 9, 2021 | Nerga. Os Castros | Vigo | Gastropod | *Littorina sp.* | TTX |
| August 10, 2021 | Aldán. Vilariño | Pontevedra | Gastropod | *Patella sp.* | TTX |
| August 10, 2021 | Aldán. Vilariño | Pontevedra | Cnidaria | *n.i.* | TTX |
| August 10, 2021 | Aldán. Vilariño | Pontevedra | Gastropod | *n.i.* | TTX |
| August 10, 2021 | Aldán. Vilariño | Pontevedra | Gastropod | *Littorina sp.* | TTX |
| August 10, 2021 | Barallobre. Rampa | Ferrol | Bivalve | *Mytilus galloprovincialis* | TTX |
| August 10, 2021 | Miño. Lombo Espiñeira | Betanzos | Bivalve | *Ruditapes philippinarum* | TTX |
| August 10, 2021 | Pasaxe. Sta Cristina | A Coruña | Bivalve | *Cerastoderma edule* | TTX |
| August 10, 2021 | Camariñas. Rio da Ponte | Camariñas | Bivalve | *Ruditapes decussatus* | TTX |
| August 11, 2021 | Barqueiro. Salgueira | Barqueiro | Bivalve | *Mytilus galloprovincialis* | TTX |
| August 11, 2021 | Corcubión. Cee | Corcubión | Bivalve | *Cerastoderma edule* | |
| August 11, 2021 | Muros I. Abelleira | Muros-Noia | Bivalve | *Cerastoderma edule* | TTX |
| August 17, 2021 | Pasaxe. Sta Cristina | A Coruña | Bivalve | *Cerastoderma edule* | TTX |
| August 17, 2021 | Corcubión. Cee | Corcubión | Bivalve | *Cerastoderma edule* | TTX |
| August 18, 2021 | Barqueiro. Salgueira | Barqueiro | Bivalve | *Mytilus galloprovincialis* | TTX |
| August 18, 2021 | Miño. Lombo Espiñeira | Betanzos | Bivalve | *Ruditapes philippinarum* | TTX |
| August 18, 2021 | Camariñas. Rio da Ponte | Camariñas | Bivalve | *Ruditapes decussatus* | TTX |
| August 18, 2021 | Muros I | Muros-Noia | Bivalve | *Cerastoderma edule* | TTX |
| August 19, 2021 | Barallobre. Rampa | Ferrol | Bivalve | *Mytilus galloprovincialis* | TTX |
| August 20, 2021 | Vilanova de Arousa. Corón | Arousa | Gastropod | *n.i.* | TTX |
| August 20, 2021 | Vilanova de Arousa. Corón | Arousa | Crustacean | *Balanus sp.* | TTX |
| August 20, 2021 | Vilanova de Arousa. Corón | Arousa | Gastropod | *Littorina sp.* | TTX |
| August 20, 2021 | Vilanova de Arousa. Corón | Arousa | Gastropod | *Littorina sp.* | TTX |
| August 20, 2021 | Vilanova de Arousa. Corón | Arousa | Gastropod | *Nucella sp.* | TTX |

| | | | | | | |
|---|---|---|---|---|---|---|
| August 20, 2021 | Vilanova de Arousa. Corón | Arousa | Cnidaria | *n.i.* | | TTX |
| August 20, 2021 | Vilanova de Arousa. Corón | Arousa | Bivalve | *Mytilus galloprovincialis* | | TTX |
| August 20, 2021 | Vilanova de Arousa. Corón | Arousa | Gastropod | *Patella sp.* | | TTX |
| August 23, 2021 | Arousa VI | Arousa | Bivalve | *Ruditapes philippinarum* | | TTX |
| August 23, 2021 | Pont II. Aldán | Pontevedra | Bivalve | *Venerupis rhomboides* | | TTX |
| August 24, 2021 | Barallobre | Ferrol | Bivalve | *Mytilus galloprovincialis* | | TTX |
| August 24, 2021 | Pasaxe. Sta Cristina | A Coruña | Bivalve | *Cerastoderma edule* | | TTX |
| August 24, 2021 | Camariñas. Rio da Ponte | Camariñas | Bivalve | *Ruditapes decussatus* | | TTX |
| August 24, 2021 | Muros I | Muros-Noia | Bivalve | *Cerastoderma edule* | | TTX |
| August 24, 2021 | Vigo II.2 | Vigo | Bivalve | *Ruditapes philippinarum* | | TTX |
| August 25, 2021 | Bueu. Beluso | Pontevedra | Echinoderm | *n.i.* | | TTX |
| August 25, 2021 | Bueu. Beluso | Pontevedra | Gastropod | *Nucella sp.* | | TTX |
| August 25, 2021 | Bueu. Beluso | Pontevedra | Gastropod | *Littorina sp.* | | TTX |
| August 25, 2021 | Bueu. Beluso | Pontevedra | Gastropod | *Crepidula sp. fornicata* | | TTX |
| August 25, 2021 | Bueu. Beluso | Pontevedra | Echinoderm | *n.i.* | | TTX |
| August 25, 2021 | Bueu. Beluso | Pontevedra | Cnidaria | *n.i.* | | TTX |
| August 25, 2021 | Bueu. Beluso | Pontevedra | Gastropod | *Patella sp.* | | TTX |
| August 25, 2021 | Bueu. Beluso | Pontevedra | Bivalve | *Mytilus galloprovincialis* | | TTX |
| August 25, 2021 | Miño. Lombo Espiñeira | Betanzos | Bivalve | *Ruditapes philippinarum* | | TTX |
| August 25, 2021 | Corcubión | Corcubión | Bivalve | *Cerastoderma edule* | | TTX |
| August 25, 2021 | Pont V. Placeres | Pontevedra | Bivalve | *Ruditapes philippinarum* | | TTX |
| August 26, 2021 | Arousa I. Meloxo | Arousa | Bivalve | *Venerupis pullastra* | | TTX |
| August 31, 2021 | Miño. Lombo Espiñeira | Betanzos | Bivalve | *Ruditapes philippinarum* | | TTX |
| August 31, 2021 | Pasaxe. Sta Cristina | A Coruña | Bivalve | *Cerastoderma edule* | | TTX |
| August 31, 2021 | Corcubión. Cee | Corcubión | Bivalve | *Cerastoderma edule* | | TTX |
| August 31, 2021 | Muros I | Muros-Noia | Bivalve | *Cerastoderma edule* | | TTX |
| August 31, 2021 | Pont II. Area Brava | Pontevedra | Bivalve | *Venerupis rhomboides* | | TTX |
| September 1, 2021 | Barallobre. Rampa | Ferrol | Bivalve | *Mytilus galloprovincialis* | | TTX |
| September 1, 2021 | Arousa. Bohido | Arousa | Bivalve | *Tellina donacina* | 5,6,11 trideoxy TTX | TTX |
| September 1, 2021 | Arousa. Bohido | Arousa | Echinoderm | *Leptosynapta sp.* | | TTX |
| September 1, 2021 | Arousa. Bohido | Arousa | Bivalve | *Scrobicularia plana* | | TTX |

| | | | | | |
|---|---|---|---|---|---|
| September 1, 2021 | Arousa. Bohido | Arousa | Gastropod | *Crepidula sp. fornicata* | TTX |
| September 1, 2021 | Arousa. Bohido | Arousa | Bivalve | *Mactra sp.* | TTX |
| September 1, 2021 | Arousa. Bohido | Arousa | Bivalve | *Gari depressa* | TTX |
| September 1, 2021 | Arousa. Bohido | Arousa | Bivalve | *Lutraria sp.* | TTX |
| September 1, 2021 | Arousa. Bohido | Arousa | Polychaete | *Sipunculus nudus* | TTX |
| September 1, 2021 | Arousa. Bohido | Arousa | Crustacean | *Atelecyclus undecimdentatus* | TTX |
| September 3, 2021 | Camariñas. Rio da Ponte | Camariñas | Bivalve | *Ruditapes philippinarum* | TTX |
| September 3, 2021 | Pont V | Pontevedra | Bivalve | *Ruditapes philippinarum* | TTX |
| September 3, 2021 | Vigo II.2 | Vigo | Bivalve | *Ruditapes philippinarum* | TTX |
| September 7, 2021 | Rianxo. Porrón | Arousa | Bivalve | *Mytilus galloprovincialis* | TTX |
| September 7, 2021 | Barqueiro. Salgueira | Barqueiro | Bivalve | *Mytilus galloprovincialis* | TTX |
| September 7, 2021 | Miño. Lombo Espiñeira | Betanzos | Bivalve | *Ruditapes philippinarum* | TTX |
| September 7, 2021 | Pasaxe. Sta Cristina | A Coruña | Bivalve | *Cerastoderma edule* | TTX |
| September 7, 2021 | Camariñas. Rio da Ponte | Camariñas | Bivalve | *Ruditapes decussatus* | TTX |
| September 7, 2021 | Muros I. Abelleira | Muros-Noia | Bivalve | *Cerastoderma edule* | TTX |
| September 7, 2021 | Muros III. Testal | Muros-Noia | Bivalve | *Cerastoderma edule* | TTX |
| September 7, 2021 | Pont V. Placeres | Pontevedra | Bivalve | *Ruditapes philippinarum* | TTX |
| September 8, 2021 | Barallobre. Rampa | Ferrol | Bivalve | *Mytilus galloprovincialis* | TTX |
| September 8, 2021 | Corcubión. Cee | Corcubión | Bivalve | *Cerastoderma edule* | TTX |
| September 13, 2021 | Muros III | Muros-Noia | Bivalve | *Cerastoderma edule* | TTX |
| September 14, 2021 | Miño. Lombo Espiñeira | Betanzos | Bivalve | *Ruditapes philippinarum* | TTX |
| September 14, 2021 | Pasaxe. Sta Cristina | A Coruña | Bivalve | *Cerastoderma edule* | TTX |
| September 14, 2021 | Corcubión. Cee | Corcubión | Bivalve | *Cerastoderma edule* | TTX |
| September 14, 2021 | Arousa I. Rons | Arousa | Bivalve | *Venerupis pullastra* | TTX |
| September 14, 2021 | Pont V. Placeres | Pontevedra | Bivalve | *Ruditapes philippinarum* | TTX |
| September 14, 2021 | Vigo II.2 Xunqueira | Vigo | Bivalve | *Ruditapes philippinarum* | TTX |
| September 15, 2021 | Barqueiro. Salgueira | Barqueiro | Bivalve | *Mytilus galloprovincialis* | TTX |
| September 15, 2021 | Barallobre. Rampa | Ferrol | Bivalve | *Mytilus galloprovincialis* | TTX |
| September 16, 2021 | Camariñas. Ariño | Camariñas | Bivalve | *Ruditapes decussatus* | TTX |
| September 16, 2021 | Vigo I. Cies | Vigo | Bivalve | *Venerupis rhomboides* | TTX |
| September 20, 2021 | Pont V. Placeres | Pontevedra | Bivalve | *Ruditapes philippinarum* | TTX |

| | | | | | |
|---|---|---|---|---|---|
| September 20, 2021 | Vigo II.2 | Vigo | Bivalve | *Ruditapes philippinarum* | TTX |
| September 21, 2021 | Barqueiro. Salgueira | Barqueiro | Bivalve | *Mytilus galloprovincialis* | TTX |
| September 21, 2021 | Miño. Lombo Espiñeira | Betanzos | Bivalve | *Ruditapes philippinarum* | TTX |
| September 21, 2021 | Pasaxe. Sta Cristina | A Coruña | Bivalve | *Cerastoderma edule* | TTX |
| September 21, 2021 | Camariñas. Enseada Vasa | Camariñas | Bivalve | *Ruditapes decussatus* | TTX |
| September 21, 2021 | Muros III | Muros-Noia | Bivalve | *Cerastoderma edule* | TTX |
| September 22, 2021 | Corcubión. Cee | Corcubión | Bivalve | *Mytilus galloprovincialis* | TTX |
| September 22, 2021 | Vigo I. Cies | Vigo | Bivalve | *Venerupis rhomboides* | TTX |
| September 23, 2021 | Vilanova de Arousa. Corón | Arousa | Gastropod | *Monodonta sp.* | TTX |
| September 23, 2021 | Vilanova de Arousa. Corón | Arousa | Gastropod | *Gibbula sp.* | TTX |
| September 23, 2021 | Vilanova de Arousa. Corón | Arousa | Gastropod | *Nucella sp.* | TTX |
| September 23, 2021 | Vilanova de Arousa. Corón | Arousa | Gastropod | *Littorina sp.* | TTX |
| September 23, 2021 | Vilanova de Arousa. Corón | Arousa | Cnidaria | *n.i.* | TTX |
| September 23, 2021 | Vilanova de Arousa. Corón | Arousa | Gastropod | *Patella sp.* | TTX |
| September 23, 2021 | Vilanova de Arousa. Corón | Arousa | Bivalve | *Mytilus galloprovincialis* | TTX |
| September 23, 2021 | Barallobre. Rampa | Ferrol | Bivalve | *Mytilus galloprovincialis* | TTX |
| September 24, 2021 | Rianxo. Salto do ladrón | Arousa | Gastropod | *Littorina sp.* | TTX |
| September 24, 2021 | Rianxo. Salto do ladrón | Arousa | Bivalve | *Mytilus galloprovincialis* | TTX |
| September 24, 2021 | Rianxo. Salto do ladrón | Arousa | Gastropod | *n.i.* | TTX |
| September 24, 2021 | Rianxo. Salto do ladrón | Arousa | Gastropod | *n.i.* | TTX |
| September 24, 2021 | Arousa VII | Arousa | Bivalve | *Ruditapes philippinarum* | TTX |
| September 27, 2021 | Muros III. Testal | Muros-Noia | Bivalve | *Cerastoderma edule* | TTX |
| September 28, 2021 | Miño. Lombo Espiñeira | Betanzos | Bivalve | *Ruditapes philippinarum* | TTX |
| September 28, 2021 | Pasaxe. Sta Cristina | A Coruña | Bivalve | *Cerastoderma edule* | TTX |
| September 28, 2021 | Muros I. Abelleira | Muros-Noia | Bivalve | *Cerastoderma edule* | TTX |
| September 28, 2021 | Pont V. Placeres | Pontevedra | Bivalve | *Ruditapes philippinarum* | TTX |
| September 28, 2021 | Vigo I. Cies | Vigo | Bivalve | *Venerupis rhomboides* | TTX |
| September 29, 2021 | Camariñas. Enseada Vasa | Camariñas | Bivalve | *Ruditapes decussatus* | TTX |
| September 29, 2021 | Corcubión. Cee | Corcubión | Bivalve | *Mytilus galloprovincialis* | TTX |
| September 30, 2021 | Barqueiro. Salgueira | Barqueiro | Bivalve | *Mytilus galloprovincialis* | TTX |
| October 1, 2021 | Arousa VII. Lombo das os | Arousa | Bivalve | *Ruditapes philippinarum* | TTX |

| | | | | | |
|---|---|---|---|---|---|
| October 1, 2021 | Vigo II.2 Xunqueira | Vigo | Bivalve | *Ruditapes philippinarum* | TTX |
| October 4, 2021 | Muros III. Testal | Muros-Noia | Bivalve | *Cerastoderma edule* | TTX |
| October 5, 2021 | Barqueiro. Salgueira | Barqueiro | Bivalve | *Mytilus galloprovincialis* | TTX |
| October 5, 2021 | Miño. Lombo Espiñeira | Betanzos | Bivalve | *Ruditapes philippinarum* | TTX |
| October 5, 2021 | Pasaxe. Sta Cristina | A Coruña | Bivalve | *Cerastoderma edule* | TTX |
| October 5, 2021 | Camariñas. Enseada Vasa | Camariñas | Bivalve | *Ruditapes decussatus* | TTX |
| October 5, 2021 | Corcubión. Cee | Corcubión | Bivalve | *Cerastoderma edule* | TTX |
| October 5, 2021 | Pont V | Pontevedra | Bivalve | *Ruditapes philippinarum* | TTX |
| October 13, 2021 | Corcubión. Cee | Corcubión | Bivalve | *Cerastoderma edule* | TTX |
| October 14, 2021 | Barqueiro. Salgueira | Barqueiro | Bivalve | *Mytilus galloprovincialis* | TTX |
| October 14, 2021 | Miño. Lombo Espiñeira | Betanzos | Bivalve | *Ruditapes philippinarum* | TTX |
| October 14, 2021 | Pasaxe. Sta Cristina | A Coruña | Bivalve | *Cerastoderma edule* | TTX |
| October 14, 2021 | Camariñas. Enseada Vasa | Camariñas | Bivalve | *Mytilus galloprovincialis* | TTX |
| October 14, 2021 | Muros III. Testal | Muros-Noia | Bivalve | *Cerastoderma edule* | |
| October 14, 2021 | Pont V. Placeres | Pontevedra | Bivalve | *Ruditapes philippinarum* | TTX |
| October 19, 2021 | Barqueiro. Salgueira | Barqueiro | Bivalve | *Mytilus galloprovincialis* | TTX |
| October 19, 2021 | Barallobre. Rampa | Ferrol | Bivalve | *Mytilus galloprovincialis* | TTX |
| October 19, 2021 | Miño. Lombo Espiñeira | Betanzos | Bivalve | *Ruditapes philippinarum* | TTX |
| October 19, 2021 | Pasaxe. Sta Cristina | A Coruña | Bivalve | *Cerastoderma edule* | TTX |
| October 19, 2021 | Camariñas. Rio da Ponte | Camariñas | Bivalve | *Ruditapes philippinarum* | TTX |
| October 19, 2021 | Corcubión. Cee | Corcubión | Bivalve | *Mytilus galloprovincialis* | TTX |
| October 19, 2021 | Muros III. Testal | Muros-Noia | Bivalve | *Cerastoderma edule* | TTX |
| October 21, 2021 | Pont V. Placeres | Pontevedra | Bivalve | *Ruditapes philippinarum* | TTX |
| October 22, 2021 | Pont II. San Cibrao | Pontevedra | Bivalve | *Ruditapes philippinarum* | TTX |
| October 26, 2021 | Barallobre. Rampa | Ferrol | Bivalve | *Mytilus galloprovincialis* | TTX |
| October 26, 2021 | Miño. Lombo Espiñeira | Betanzos | Bivalve | *Cerastoderma edule* | TTX |
| October 26, 2021 | Pasaxe. Sta Cristina | A Coruña | Bivalve | *Cerastoderma edule* | TTX |
| October 26, 2021 | Camariñas. Enseada Vasa | Camariñas | Bivalve | *Mytilus galloprovincialis* | TTX |
| October 26, 2021 | Corcubión. Cee | Corcubión | Bivalve | *Mytilus galloprovincialis* | TTX |
| October 27, 2021 | Barqueiro. Salgueira | Barqueiro | Bivalve | *Mytilus galloprovincialis* | TTX |
| October 27, 2021 | Pont V | Pontevedra | Bivalve | *Ruditapes philippinarum* | TTX |

| | | | | | | |
|---|---|---|---|---|---|---|
| October 27, 2021 | Vigo II.2 Xunqueira | Vigo | Bivalve | *Ruditapes philippinarum* | | TTX |
| October 29, 2021 | Ria de Arousa | Arousa | Polychaete | *Aphrodita aculeata* | | TTX |
| October 29, 2021 | Vigo I. Limens | Vigo | Bivalve | *Ruditapes philippinarum* | | TTX |
| November 3, 2021 | Barqueiro. Salgueira | Barqueiro | Bivalve | *Mytilus galloprovincialis* | | TTX |
| November 3, 2021 | Miño. Lombo Espiñeira | Betanzos | Bivalve | *Cerastoderma edule* | | TTX |
| November 3, 2021 | Pasaxe. Sta Cristina | A Coruña | Bivalve | *Cerastoderma edule* | | TTX |
| November 3, 2021 | Camariñas. Enseada Vasa | Camariñas | Bivalve | *Mytilus galloprovincialis* | | TTX |
| November 3, 2021 | Muros III. Testal | Muros-Noia | Bivalve | *Cerastoderma edule* | | TTX |
| November 3, 2021 | Vigo I. Liméns | Vigo | Bivalve | *Venerupis rhomboides* | | TTX |
| November 3, 2021 | Vigo II.2. Tirán | Vigo | Bivalve | *Venerupis rhomboides* | | TTX |
| November 4, 2021 | Barallobre. Rampa | Ferrol | Bivalve | *Mytilus galloprovincialis* | | TTX |
| November 4, 2021 | Corcubión. Cee | Corcubión | Bivalve | *Mytilus galloprovincialis* | | TTX |
| November 5, 2021 | Ramallosa. Esteiro Foz. | Vigo | Bivalve | *Ruditapes philippinarum* | | TTX |
| November 5, 2021 | Ramallosa. Esteiro Foz. | Vigo | Gastropod | *Littorina sp.* | | TTX |
| November 5, 2021 | Ramallosa. Esteiro Foz. | Vigo | Gastropod | *Patella sp.* | | TTX |
| November 5, 2021 | Ramallosa. Esteiro Foz. | Vigo | Bivalve | *Mytilus galloprovincialis* | | TTX |
| November 5, 2021 | Ramallosa. Esteiro Foz. | Vigo | Cnidaria | *n.i.* | 5,6,11 trideoxy TTX | TTX |
| November 5, 2021 | Ramallosa. Esteiro Foz. | Vigo | Bivalve | *Ostrea edulis* | | TTX |
| November 7, 2021 | Rianxo. Porrón | Arousa | Crustacean | *Polybius sp.* | | TTX |
| November 7, 2021 | Rianxo. Porrón | Arousa | Bivalve | *Cerastoderma edule* | | TTX |
| November 7, 2021 | Rianxo. Porrón | Arousa | Echinoderm | *Asterina sp.* | | TTX |
| November 7, 2021 | Rianxo. Porrón | Arousa | Bivalve | *Mytilus galloprovincialis* | | TTX |
| November 7, 2021 | Rianxo. Porrón | Arousa | Gastropod | *n.i.* | | TTX |
| November 7, 2021 | Rianxo. Porrón | Arousa | Poriferous | *n.i.* | | TTX |
| November 7, 2021 | Rianxo. Porrón | Arousa | Crustacean | *n.i.* | | TTX |
| November 8, 2021 | Porto do Son. Arnela | Muros-Noia | Gastropod | *Monodonta sp.* | | TTX |
| November 8, 2021 | Porto do Son. Arnela | Muros-Noia | Gastropod | *Gibbula sp.* | | TTX |
| November 8, 2021 | Porto do Son. Arnela | Muros-Noia | Gastropod | *Nucella sp.* | | TTX |
| November 8, 2021 | Porto do Son. Arnela | Muros-Noia | Gastropod | *Ocenebra erinacea* | | TTX |
| November 8, 2021 | Porto do Son. Arnela | Muros-Noia | Polychaete | *n.i.* | | TTX |
| November 8, 2021 | Porto do Son. Arnela | Muros-Noia | Crustacean | *Balanus sp.* | | TTX |

| | | | | | |
|---|---|---|---|---|---|
| November 8, 2021 | Porto do Son. Arnela | Muros-Noia | Echinoderm | *Asterina sp.* | TTX |
| November 8, 2021 | Porto do Son. Arnela | Muros-Noia | Cnidaria | *n.i.* | TTX |
| November 8, 2021 | Porto do Son. Arnela | Muros-Noia | Gastropod | *Patella sp.* | TTX |
| November 8, 2021 | Porto do Son. Arnela | Muros-Noia | Gastropod | *Patella sp.* | TTX |
| November 8, 2021 | Porto do Son. Arnela | Muros-Noia | Gastropod | *Patella sp.* | TTX |
| November 8, 2021 | Porto do Son. Arnela | Muros-Noia | Bivalve | *Mytilus galloprovincialis* | TTX |
| November 8, 2021 | Porto do Son. Arnela | Muros-Noia | Bivalve | *Mytilus galloprovincialis* | TTX |
| November 8, 2021 | Pont V. Placeres | Pontevedra | Gastropod | *Littorina sp.* | TTX |
| November 8, 2021 | Pont V. Placeres | Pontevedra | Gastropod | *Patella sp.* | TTX |
| November 8, 2021 | Pont V. Placeres | Pontevedra | Crustacean | *Polybius sp.* | TTX |
| November 8, 2021 | Pont V. Placeres | Pontevedra | Bivalve | *Ostrea edulis* | TTX |
| November 8, 2021 | Pont V. Placeres | Pontevedra | Bivalve | *Ruditapes philippinarum* | TTX |
| November 8, 2021 | Pont V. Placeres | Pontevedra | Crustacean | *Balanus sp.* | TTX |
| November 8, 2021 | Pont V. Placeres | Pontevedra | Gastropod | *Nassarius sp.* | |
| November 8, 2021 | Pont V. Placeres | Pontevedra | Bivalve | *Cerastoderma edule* | TTX |
| November 8, 2021 | Pont V. Placeres | Pontevedra | Bivalve | *Mytilus galloprovincialis* | TTX |
| November 9, 2021 | Rianxo | Arousa | Gastropod | *Crepidula sp.* | TTX |
| November 9, 2021 | Rianxo | Arousa | Bivalve | *Aequipecten opercularis* | TTX |
| November 9, 2021 | Rianxo | Arousa | Bivalve | *Pecten maximus* | TTX |
| November 9, 2021 | Rianxo | Arousa | Bivalve | *Chlamys varia* | TTX |
| November 9, 2021 | Barqueiro. Salgueira | Barqueiro | Bivalve | *Mytilus galloprovincialis* | TTX |
| November 9, 2021 | Barallobre. Rampa | Ferrol | Bivalve | *Ruditapes philippinarum* | TTX |
| November 9, 2021 | Miño. Lombo Espiñeira | Betanzos | Bivalve | *Cerastoderma edule* | TTX |
| November 9, 2021 | Pasaxe. Sta Cristina | A Coruña | Bivalve | *Cerastoderma edule* | TTX |
| November 9, 2021 | Camariñas. Río da Ponte | Camariñas | Bivalve | *Mytilus galloprovincialis* | TTX |
| November 9, 2021 | Pont V. Placeres | Pontevedra | Bivalve | *Ruditapes philippinarum* | TTX |
| November 10, 2021 | Vigo I | Vigo | Bivalve | *Venerupis rhomboides* | TTX |
| November 11, 2021 | Corcubión. Cee | Corcubión | Bivalve | *Mytilus galloprovincialis* | TTX |
| November 11, 2021 | Vigo II.2 Tirán | Vigo | Bivalve | *Venerupis rhomboides* | TTX |
| November 16, 2021 | Barallobre. Rampa | Ferrol | Bivalve | *Mytilus galloprovincialis* | TTX |
| November 16, 2021 | Pasaxe. Sta Cristina | A Coruña | Bivalve | *Cerastoderma edule* | TTX |

| | | | | | |
|---|---|---|---|---|---|
| November 16, 2021 | Camariñas. Río da ponte | Camariñas | Bivalve | *Mytilus galloprovincialis* | TTX |
| November 16, 2021 | Corcubión. Cee | Corcubión | Bivalve | *Mytilus galloprovincialis* | TTX |
| November 17, 2021 | Barqueiro. Salgueira | Barqueiro | Bivalve | *Mytilus galloprovincialis* | TTX |
| November 17, 2021 | Miño. Muro Petra Sabío | Betanzos | Bivalve | *Mytilus galloprovincialis* | TTX |
| November 23, 2021 | Barallobre. Rampa | Ferrol | Bivalve | *Mytilus galloprovincialis* | TTX |
| November 23, 2021 | Miño. Muro Petra Sabío | Betanzos | Bivalve | *Mytilus galloprovincialis* | TTX |
| November 23, 2021 | Pasaxe. Sta Cristina | A Coruña | Bivalve | *Cerastoderma edule* | TTX |
| November 23, 2021 | Camariñas. Enseada Vasa | Camariñas | Bivalve | *Mytilus galloprovincialis* | TTX |
| November 23, 2021 | Corcubión. Cee | Corcubión | Bivalve | *Mytilus galloprovincialis* | TTX |
| November 24, 2021 | Barqueiro. Salgueira | Barqueiro | Bivalve | *Mytilus galloprovincialis* | TTX |
| November 26, 2021 | Vigo I | Vigo | Bivalve | *Venerupis rhomboides* | TTX |
| November 30, 2021 | Carnota. Lira | Corcubión | Echinoderm | *Paracentrotus sp.* | TTX |
| November 30, 2021 | Barqueiro. Salgueira | Barqueiro | Bivalve | *Mytilus galloprovincialis* | TTX |
| November 30, 2021 | Barallobre. Rampa | Ferrol | Bivalve | *Mytilus galloprovincialis* | TTX |
| November 30, 2021 | Miño. Muro Petra Sabío | Betanzos | Bivalve | *Mytilus galloprovincialis* | TTX |
| November 30, 2021 | Pasaxe. Sta Cristina | A Coruña | Bivalve | *Cerastoderma edule* | TTX |
| November 30, 2021 | Corcubión. Cee | Corcubión | Bivalve | *Mytilus galloprovincialis* | TTX |
| December 2, 2021 | Camariñas. Enseada Vasa | Camariñas | Bivalve | *Mytilus galloprovincialis* | TTX |
| December 9, 2021 | Barqueiro. Salgueira | Barqueiro | Bivalve | *Mytilus galloprovincialis* | TTX |
| December 9, 2021 | Barallobre. Rampa | Ferrol | Bivalve | *Mytilus galloprovincialis* | TTX |
| December 9, 2021 | Miño. Muro Petra Sabío | Betanzos | Bivalve | *Mytilus galloprovincialis* | TTX |
| December 9, 2021 | Pasaxe. Sta Cristina | A Coruña | Bivalve | *Cerastoderma edule* | TTX |
| December 9, 2021 | Camariñas. Enseada Vasa | Camariñas | Bivalve | *Mytilus galloprovincialis* | TTX |
| December 10, 2021 | Corcubión. Cee | Corcubión | Bivalve | *Mytilus galloprovincialis* | TTX |
| December 14, 2021 | Barallobre. Rampa | Ferrol | Bivalve | *Mytilus galloprovincialis* | TTX |
| December 14, 2021 | Miño. Muro Petra Sabío | Betanzos | Bivalve | *Mytilus galloprovincialis* | TTX |
| December 14, 2021 | Pasaxe. Sta Cristina | A Coruña | Bivalve | *Cerastoderma edule* | TTX |
| December 15, 2021 | Barqueiro. Salgueira | Barqueiro | Bivalve | *Mytilus galloprovincialis* | TTX |
| December 15, 2021 | Corcubión. Cee | Corcubión | Bivalve | *Mytilus galloprovincialis* | TTX |
| December 16, 2021 | Camariñas. Enseada Vasa | Camariñas | Bivalve | *Mytilus galloprovincialis* | TTX |
| December 21, 2021 | Barqueiro. Salgueira | Barqueiro | Bivalve | *Mytilus galloprovincialis* | TTX |

| | | | | | |
|---|---|---|---|---|---|
| December 21, 2021 | Barallobre.  Rampa | Ferrol | Bivalve | *Mytilus galloprovincialis* | TTX |
| December 21, 2021 | Miño.  Muro Petra Sabío | Betanzos | Bivalve | *Mytilus galloprovincialis* | TTX |
| December 21, 2021 | Camariñas. Enseada Vasa | Camariñas | Bivalve | *Mytilus galloprovincialis* | TTX |
| December 22, 2021 | Pasaxe. Sta Cristina | A Coruña | Bivalve | *Cerastoderma edule* | TTX |
| December 23, 2021 | Corcubión. Cee | Corcubión | Bivalve | *Mytilus galloprovincialis* | TTX |
| December 28, 2021 | Barallobre.  Rampa | Ferrol | Bivalve | *Mytilus galloprovincialis* | TTX |
| December 28, 2021 | Miño. Muro Petra Sabío | Betanzos | Bivalve | *Mytilus galloprovincialis* | TTX |
| December 28, 2021 | Pasaxe. Sta Cristina | A Coruña | Bivalve | *Cerastoderma edule* | TTX |
| December 28, 2021 | Camariñas. Río da Ponte | Camariñas | Bivalve | *Mytilus galloprovincialis* | TTX |
| December 28, 2021 | Corcubión. Cee | Corcubión | Bivalve | *Mytilus galloprovincialis* | TTX |
| December 29, 2021 | Barqueiro. Salgueira | Barqueiro | Bivalve | *Mytilus galloprovincialis* | TTX |
| January 4, 2022 | Barqueiro. Salgueira | Barqueiro | Bivalve | *Mytilus galloprovincialis* | TTX |
| January 4, 2022 | Barallobre. Pantalan | Ferrol | Bivalve | *Mytilus galloprovincialis* | TTX |
| January 4, 2022 | Miño. Muro Petra Sabío | Betanzos | Bivalve | *Mytilus galloprovincialis* | TTX |
| January 4, 2022 | Pasaxe. Sta Cristina | A Coruña | Bivalve | *Cerastoderma edule* | TTX |
| January 4, 2022 | Camariñas. Enseada Vasa | Camariñas | Bivalve | *Mytilus galloprovincialis* | TTX |
| January 5, 2022 | Corcubión. Cee | Corcubión | Bivalve | *Mytilus galloprovincialis* | TTX |
| January 11, 2022 | Barallobre. Rampa | Ferrol | Bivalve | *Mytilus galloprovincialis* | TTX |
| January 11, 2022 | Miño. Muro Petra Sabío | Betanzos | Bivalve | *Mytilus galloprovincialis* | TTX |
| January 11, 2022 | Pasaxe. Sta Cristina | A Coruña | Bivalve | *Cerastoderma edule* | TTX |
| January 11, 2022 | Camariñas. Enseada Vasa | Camariñas | Bivalve | *Mytilus galloprovincialis* | TTX |
| January 11, 2022 | Corcubión. Cee | Corcubión | Bivalve | *Mytilus galloprovincialis* | TTX |
| January 12, 2022 | Barqueiro. Salgueira | Barqueiro | Bivalve | *Mytilus galloprovincialis* | TTX |
| January 18, 2022 | Miño. Muro Petra Sabío | Betanzos | Bivalve | *Mytilus galloprovincialis* | TTX |
| January 18, 2022 | Pasaxe. Sta Cristina | A Coruña | Bivalve | *Mytilus galloprovincialis* | TTX |
| January 18, 2022 | Camariñas. Enseada Vasa | Camariñas | Bivalve | *Mytilus galloprovincialis* | TTX |
| January 18, 2022 | Corcubión. Cee | Corcubión | Bivalve | *Mytilus galloprovincialis* | TTX |
| January 19, 2022 | Barallobre. Rampa | Ferrol | Bivalve | *Mytilus galloprovincialis* | TTX |
| January 20, 2022 | Vilanova de Arousa. As Sinas | Arousa | Cnidaria | *Actinia sp.* | TTX |
| January 20, 2022 | Vilanova de Arousa. As Sinas | Arousa | Cnidaria | *Actinia sp.* | TTX |
| January 20, 2022 | Vilanova de Arousa. As Sinas | Arousa | Gastropod | *Monodonta sp.* | TTX |

| | | | | | | |
|---|---|---|---|---|---|---|
| January 20, 2022 | Vilanova de Arousa. As Sinas | Arousa | Gastropod | *Littorina sp.* | | TTX |
| January 20, 2022 | Vilanova de Arousa. As Sinas | Arousa | Gastropod | *Gibbula sp.* | | TTX |
| January 20, 2022 | Vilanova de Arousa. As Sinas | Arousa | Gastropod | *Nucella sp.* | | TTX |
| January 20, 2022 | Vilanova de Arousa. As Sinas | Arousa | Gastropod | *Patella sp.* | | TTX |
| January 20, 2022 | Vilanova de Arousa. As Sinas | Arousa | Crustacean | *Balanus sp.* | | TTX |
| January 20, 2022 | Vilanova de Arousa. As Sinas | Arousa | Bivalve | *Mytilus galloprovincialis* | | TTX |
| January 20, 2022 | Barqueiro. Salgueira | Barqueiro | Bivalve | *Mytilus galloprovincialis* | | TTX |
| January 21, 2022 | Vilanova de Arousa. Corón | Arousa | Gastropod | *n.i.* | | TTX |
| January 21, 2022 | Vilanova de Arousa. Corón | Arousa | Crustacean | *Balanus sp.* | | TTX |
| January 21, 2022 | Vilanova de Arousa. Corón | Arousa | Gastropod | *Patella sp.* | | TTX |
| January 21, 2022 | Vilanova de Arousa. Corón | Arousa | Cnidaria | *Actinia sp.* | | TTX |
| January 21, 2022 | Vilanova de Arousa. Corón | Arousa | Bivalve | *Mytilus galloprovincialis* | | TTX |
| January 21, 2022 | Vilanova de Arousa. Corón | Arousa | Gastropod | *Nucella sp.* | | TTX |
| January 21, 2022 | Vilanova de Arousa. Corón | Arousa | Gastropod | *Monodonta sp.* | | TTX |
| January 21, 2022 | Vilanova de Arousa. Corón | Arousa | Gastropod | *Littorina sp.* | | TTX |
| January 25, 2022 | Barallobre. Rampa | Ferrol | Bivalve | *Mytilus galloprovincialis* | | TTX |
| January 25, 2022 | Miño. Muro Petra Sabío | Betanzos | Bivalve | *Mytilus galloprovincialis* | | TTX |
| January 25, 2022 | Pasaxe. Sta Cristina | A Coruña | Bivalve | *Cerastoderma edule* | | TTX |
| January 25, 2022 | Camariñas. Enseada vasa | Camariñas | Bivalve | *Mytilus galloprovincialis* | | TTX |
| January 26, 2022 | Corcubión. Cee | Corcubión | Bivalve | *Mytilus galloprovincialis* | | TTX |
| January 27, 2022 | Barqueiro. Salgueira | Barqueiro | Bivalve | *Mytilus galloprovincialis* | | TTX |
| February 1, 2022 | Barqueiro.  Salgueira | Barqueiro | Bivalve | *Mytilus galloprovincialis* | GYMs | TTX |
| February 1, 2022 | Barallobre. Rampa | Ferrol | Bivalve | *Mytilus galloprovincialis* | GYMs | TTX |
| February 1, 2022 | Miño. Muro Petra Sabío | Betanzos | Bivalve | *Mytilus galloprovincialis* | GYMs | TTX |
| February 1, 2022 | Pasaxe. Sta Cristina | A Coruña | Bivalve | *Cerastoderma edule* | GYMs | TTX |
| February 1, 2022 | Camariñas. Enseada Vasa | Camariñas | Bivalve | *Mytilus galloprovincialis* | GYMs | TTX |
| February 1, 2022 | Corcubión. Cee | Corcubión | Bivalve | *Mytilus galloprovincialis* | GYMs | TTX |
| February 4, 2022 | Carnota. Lira | Corcubión | Echinoderm | *Paracentrotus sp.* | | TTX |
| February 8, 2022 | Barallobre. Rampa | Ferrol | Bivalve | *Mytilus galloprovincialis* | GYMs | TTX |
| February 8, 2022 | Miño. Muro Petra Sabío | Betanzos | Bivalve | *Mytilus galloprovincialis* | GYMs | TTX |
| February 8, 2022 | Pasaxe. Sta Cristina | A Coruña | Bivalve | *Cerastoderma edule* | GYMs | TTX |

| | | | | | | |
|---|---|---|---|---|---|---|
| February 8, 2022 | Camariñas. Enseada Vasa | Camariñas | Bivalve | *Mytilus galloprovincialis* | GYMs | TTX |
| February 8, 2022 | Corcubión. Cee | Corcubión | Bivalve | *Mytilus galloprovincialis* | GYMs | TTX |
| February 9, 2022 | Barqueiro. Salgueira | Barqueiro | Bivalve | *Mytilus galloprovincialis* | GYMs | TTX |
| February 9, 2022 | Muros III | Muros-Noia | Bivalve | *Cerastoderma edule* | GYMs | TTX |
| February 14, 2022 | Muros III | Muros-Noia | Bivalve | *Cerastoderma edule* | GYMs | TTX |
| February 15, 2022 | Barqueiro.  Salgueira | Barqueiro | Bivalve | *Mytilus galloprovincialis* | GYMs | TTX |
| February 15, 2022 | Barallobre. Rampa | Ferrol | Bivalve | *Mytilus galloprovincialis* | GYMs | TTX |
| February 15, 2022 | Miño. Muro Petra Sabío | Betanzos | Bivalve | *Mytilus galloprovincialis* | GYMs | TTX |
| February 15, 2022 | Pasaxe. Sta Cristina | A Coruña | Bivalve | *Cerastoderma edule* | GYMs | TTX |
| February 15, 2022 | Camariñas. Enseada Vasa | Camariñas | Bivalve | *Mytilus galloprovincialis* | GYMs | TTX |
| February 15, 2022 | Corcubión. Cee | Corcubión | Bivalve | *Mytilus galloprovincialis* | GYMs | TTX |
| February 15, 2022 | Pont V.  Placeres | Pontevedra | Bivalve | *Ruditapes philippinarum* | GYMs | TTX |
| February 21, 2022 | Arousa I. | Arousa | Bivalve | *Aequipecten opercularis* | GYMs | TTX |
| February 22, 2022 | Miño. Muro Petra Sabío | Betanzos | Bivalve | *Mytilus galloprovincialis* | GYMs | TTX |
| February 22, 2022 | Pasaxe. Sta Cristina | A Coruña | Bivalve | *Cerastoderma edule* | GYMs | TTX |
| February 22, 2022 | Camariñas.  Enseada Vasa | Camariñas | Bivalve | *Mytilus galloprovincialis* | GYMs | TTX |
| February 22, 2022 | Corcubión. Cee | Corcubión | Bivalve | *Mytilus galloprovincialis* | GYMs | TTX |
| February 22, 2022 | Muros III. Testal | Muros-Noia | Bivalve | *Cerastoderma edule* | GYMs | TTX |
| February 23, 2022 | Barqueiro. Salgueira | Barqueiro | Bivalve | *Mytilus galloprovincialis* | GYMs | TTX |
| February 23, 2022 | Barallobre.  Rampa | Ferrol | Bivalve | *Mytilus galloprovincialis* | GYMs | TTX |
| March 1, 2022 | Barqueiro. Salgueira | Barqueiro | Bivalve | *Mytilus galloprovincialis* | GYMs | TTX |
| March 1, 2022 | Miño. Muro Petra Sabío | Betanzos | Bivalve | *Mytilus galloprovincialis* | GYMs | TTX |
| March 1, 2022 | Pasaxe. Sta Cristina | A Coruña | Bivalve | *Cerastoderma edule* | GYMs | TTX |
| March 1, 2022 | Camariñas. Enseada Vasa | Camariñas | Bivalve | *Mytilus galloprovincialis* | GYMs | TTX |
| March 2, 2022 | Carnota. Lira | Corcubión | Echinoderm | *Paracentrotus sp.* | GYMs | TTX |
| March 2, 2022 | Pont V. Placeres | Pontevedra | Gastropod | *Littorina sp.* | GYMs | TTX |
| March 2, 2022 | Pont V. Placeres | Pontevedra | Bivalve | *Ostrea edulis* | GYMs | TTX |
| March 2, 2022 | Pont V. Placeres | Pontevedra | Gastropod | *Patella sp.* | GYMs | TTX |
| March 2, 2022 | Pont V. Placeres | Pontevedra | Bivalve | *Ruditapes philippinarum* | GYMs | TTX |
| March 2, 2022 | Pont V. Placeres | Pontevedra | Bivalve | *Mytilus galloprovincialis* | GYMs | TTX |
| March 2, 2022 | Barallobre. Rampa | Ferrol | Bivalve | *Mytilus galloprovincialis* | GYMs | TTX |

| | | | | | | | |
|---|---|---|---|---|---|---|---|
| March 2, 2022 | Corcubión. Cee | Corcubión | Bivalve | *Cerastoderma edule* | GYMs | | TTX |
| March 4, 2022 | Vilanova de Arousa. Corón | Arousa | Bivalve | *Cerastoderma edule* | GYMs | | TTX |
| March 4, 2022 | Vilanova de Arousa. Corón | Arousa | Gastropod | *n.i.* | GYMs | | TTX |
| March 4, 2022 | Vilanova de Arousa. Corón | Arousa | Crustacean | *Polybius sp.* | GYMs | | TTX |
| March 4, 2022 | Vilanova de Arousa. Corón | Arousa | Bivalve | *Mytilus galloprovincialis* | GYMs | | TTX |
| March 4, 2022 | Vilanova de Arousa. Corón | Arousa | Gastropod | *Littorina sp.* | GYMs | | TTX |
| March 4, 2022 | Vilanova de Arousa. Corón | Arousa | Gastropod | *Nucella sp.* | GYMs | | TTX |
| March 4, 2022 | Vilanova de Arousa. Corón | Arousa | Poriferous | *n.i.* | GYMs | | TTX |
| March 4, 2022 | Vilanova de Arousa. Corón | Arousa | Gastropod | *Nucella sp.* | GYMs | | TTX |
| March 4, 2022 | Vilanova de Arousa. Corón | Arousa | Gastropod | *Patella sp.* | GYMs | | TTX |
| March 4, 2022 | Vilanova de Arousa. Corón | Arousa | Gastropod | *Nassarius sp.* | GYMs | | TTX |
| March 4, 2022 | Vilanova de Arousa. Corón | Arousa | Bivalve | *Ruditapes philippinarum* | GYMs | | TTX |
| March 4, 2022 | Vilanova de Arousa. Corón | Arousa | Gastropod | *Monodonta sp.* | GYMs | | TTX |
| March 4, 2022 | Vilanova de Arousa. Corón | Arousa | Crustacean | *Balanus sp.* | GYMs | | TTX |
| March 4, 2022 | Vilanova de Arousa. Corón | Arousa | Cnidaria | *n.i.* | GYMs | | TTX |
| March 4, 2022 | Vilanova de Arousa. Corón | Arousa | Cnidaria | *n.i.* | | | TTX |
| March 4, 2022 | Porto do Son. Arnela | Muros-Noia | Bivalve | *Mytilus galloprovincialis* | GYMs | | TTX |
| March 4, 2022 | Porto do Son. Arnela | Muros-Noia | Gastropod | *Patella sp.* | GYMs | | TTX |
| March 4, 2022 | Porto do Son. Arnela | Muros-Noia | Crustacean | *Balanus sp.* | GYMs | | TTX |
| March 4, 2022 | Porto do Son. Arnela | Muros-Noia | Cnidaria | *n.i.* | GYMs | | TTX |
| March 4, 2022 | Porto do Son. Arnela | Muros-Noia | Cnidaria | *n.i.* | GYMs | 5,6,11 trideoxy TTX | TTX |
| March 4, 2022 | Porto do Son. Arnela | Muros-Noia | Polychaete | *n.i.* | GYMs | 5,6,11 trideoxy TTX | TTX |
| March 4, 2022 | Porto do Son. Arnela | Muros-Noia | Gastropod | *Monodonta sp.* | GYMs | | TTX |
| March 4, 2022 | Porto do Son. Arnela | Muros-Noia | Gastropod | *Gibbula sp.* | GYMs | | TTX |
| March 4, 2022 | Porto do Son. Arnela | Muros-Noia | Gastropod | *Nucella sp.* | GYMs | | TTX |
| March 8, 2022 | Barqueiro. Salgueira | Barqueiro | Bivalve | *Mytilus galloprovincialis* | GYMs | | TTX |
| March 8, 2022 | Miño. Muro Petra Sabío | Betanzos | Bivalve | *Mytilus galloprovincialis* | GYMs | | TTX |
| March 8, 2022 | Pasaxe. Sta Cristina | A Coruña | Bivalve | *Cerastoderma edule* | GYMs | | TTX |
| March 8, 2022 | Camariñas. Enseada Vasa | Camariñas | Bivalve | *Mytilus galloprovincialis* | GYMs | | TTX |
| March 8, 2022 | Corcubión. Cee | Corcubión | Bivalve | *Mytilus galloprovincialis* | GYMs | | TTX |
| March 9, 2022 | Barallobre. Rampa | Ferrol | Bivalve | *Mytilus galloprovincialis* | GYMs | | TTX |

| | | | | | | |
|---|---|---|---|---|---|---|
| March 15, 2022 | Barallobre. Rampa | Ferrol | Bivalve | *Mytilus galloprovincialis* | GYMs | TTX |
| March 17, 2022 | Miño. Muro Petra Sabío | Betanzos | Bivalve | *Mytilus galloprovincialis* | GYMs | TTX |
| March 17, 2022 | Camariñas. Enseada Vasa | Camariñas | Bivalve | *Mytilus galloprovincialis* | GYMs | TTX |
| March 22, 2022 | Miño. Muro Petra Sabío | Betanzos | Bivalve | *Mytilus galloprovincialis* | GYMs | TTX |
| March 22, 2022 | Camariñas. Enseada Vasa | Camariñas | Bivalve | *Mytilus galloprovincialis* | GYMs | TTX |
| March 23, 2022 | Corcubión. Cee | Corcubión | Bivalve | *Mytilus galloprovincialis* | GYMs | TTX |
| March 24, 2022 | Barqueiro. Salgueira | Barqueiro | Bivalve | *Mytilus galloprovincialis* | GYMs | TTX |
| March 25, 2022 | Barallobre. Rampa | Ferrol | Bivalve | *Mytilus galloprovincialis* | GYMs | TTX |
| March 29, 2022 | Barallobre. Rampa | Ferrol | Bivalve | *Mytilus galloprovincialis* | GYMs | TTX |
| March 29, 2022 | Miño. Muro Petra Sabío | Betanzos | Bivalve | *Mytilus galloprovincialis* | GYMs | TTX |
| March 29, 2022 | Pasaxe. Sta Cristina | A Coruña | Bivalve | *Cerastoderma edule* | GYMs | TTX |
| March 29, 2022 | Camariñas. Enseada Vasa | Camariñas | Bivalve | *Mytilus galloprovincialis* | GYMs | TTX |
| March 30, 2022 | Barqueiro. Salgueira | Barqueiro | Bivalve | *Mytilus galloprovincialis* | GYMs | TTX |
| March 30, 2022 | Muros III | Muros-Noia | Bivalve | *Cerastoderma edule* | GYMs | TTX |
| March 31, 2022 | Corcubión. Cee | Corcubión | Bivalve | *Mytilus galloprovincialis* | GYMs | TTX |
| April 4, 2022 | Muros III. Testal | Muros-Noia | Bivalve | *Cerastoderma edule* | GYMs | TTX |
| April 5, 2022 | Barqueiro. Salgueira | Barqueiro | Bivalve | *Mytilus galloprovincialis* | GYMs | TTX |
| April 5, 2022 | Barallobre. Rampa | Ferrol | Bivalve | *Mytilus galloprovincialis* | GYMs | TTX |
| April 5, 2022 | Miño. Muro Petra Sabío | Betanzos | Bivalve | *Mytilus galloprovincialis* | GYMs | TTX |
| April 5, 2022 | Pasaxe. Sta Cristina | A Coruña | Bivalve | *Cerastoderma edule* | GYMs | TTX |
| April 5, 2022 | Camariñas. Enseada Vasa | Camariñas | Bivalve | *Mytilus galloprovincialis* | GYMs | TTX |
| April 5, 2022 | Muros I | Muros-Noia | Bivalve | *Ensis ensis* | GYMs | TTX |
| April 5, 2022 | Pont II | Pontevedra | Bivalve | *Ensis ensis* | GYMs | TTX |
| April 6, 2022 | Arousa. Bohido | Arousa | Bivalve | *Venus verrucosa* | GYMs | TTX |
| April 6, 2022 | Arousa. Bohido | Arousa | Bivalve | *Dosinia exoleta* | GYMs | TTX |
| April 6, 2022 | Camariñas. Enseada Vasa | Camariñas | Bivalve | *Ruditapes decussatus* | GYMs | TTX |
| April 6, 2022 | Corcubión. Cee | Corcubión | Bivalve | *Mytilus galloprovincialis* | GYMs | TTX |
| April 6, 2022 | Muros I | Muros-Noia | Bivalve | *Cerastoderma edule* | GYMs | TTX |
| April 6, 2022 | Muros III | Muros-Noia | Bivalve | *Cerastoderma edule* | GYMs | TTX |
| April 8, 2022 | Nigrán. Patos | Vigo | Gastropod | *Monodonta sp.* | GYMs | TTX |
| April 8, 2022 | Nigrán. Patos | Vigo | Gastropod | *Patella sp.* | GYMs | TTX |

| | | | | | | |
|---|---|---|---|---|---|---|
| April 8, 2022 | Nigrán. Patos | Vigo | Gastropod | *n.i.* | GYMs | TTX |
| April 11, 2022 | Muros III. Testal | Muros-Noia | Bivalve | *Cerastoderma edule* | GYMs | TTX |
| April 12, 2022 | Barallobre. Rampa | Ferrol | Bivalve | *Cerastoderma edule* | GYMs | TTX |
| April 12, 2022 | Miño. Lombo Espiñeira | Betanzos | Bivalve | *Cerastoderma edule* | GYMs | TTX |
| April 12, 2022 | Pasaxe. Sta Cristina | A Coruña | Bivalve | *Cerastoderma edule* | GYMs | TTX |
| April 12, 2022 | Muros I. Cabeiro | Muros-Noia | Bivalve | *Ensis ensis* | GYMs | TTX |
| April 13, 2022 | Barqueiro. Salgueira | Barqueiro | Bivalve | *Cerastoderma edule* | GYMs | TTX |
| April 13, 2022 | Camariñas. Enseada Vasa | Camariñas | Bivalve | *Ruditapes decussatus* | GYMs | TTX |
| April 13, 2022 | Corcubión. Cee | Corcubión | Bivalve | *Cerastoderma edule* | GYMs | TTX |
| April 13, 2022 | Muros I. Abelleira | Muros-Noia | Bivalve | *Cerastoderma edule* | GYMs | TTX |
| April 18, 2022 | Porto do Son. Arnela | Muros-Noia | Gastropod | *Nucella sp.* | GYMs | TTX |
| April 18, 2022 | Porto do Son. Arnela | Muros-Noia | Gastropod | *Monodonta sp.* | GYMs | TTX |
| April 18, 2022 | Porto do Son. Arnela | Muros-Noia | Gastropod | *Gibbula sp.* | GYMs | TTX |
| April 18, 2022 | Porto do Son. Arnela | Muros-Noia | Echinoderm | *Asterina sp.* | GYMs | TTX |
| April 18, 2022 | Porto do Son. Arnela | Muros-Noia | Gastropod | *Patella sp.* | GYMs | TTX |
| April 18, 2022 | Porto do Son. Arnela | Muros-Noia | Echinoderm | *Paracentrotus sp.* | GYMs | TTX |
| April 18, 2022 | Porto do Son. Arnela | Muros-Noia | Cnidaria | *n.i.* | GYMs | TTX |
| April 18, 2022 | Porto do Son. Arnela | Muros-Noia | Bivalve | *Mytilus galloprovincialis* | GYMs | TTX |
| April 18, 2022 | Porto do Son. Arnela | Muros-Noia | Polychaete | *n.i.* | GYMs | TTX |
| April 18, 2022 | Muros III. Testal | Muros-Noia | Bivalve | *Cerastoderma edule* | GYMs | TTX |
| April 19, 2022 | Vilanova de Arousa. Corón | Arousa | Crustacean | *Polybius sp.* | GYMs | TTX |
| April 19, 2022 | Vilanova de Arousa. Corón | Arousa | Bivalve | *Cerastoderma edule* | GYMs | TTX |
| April 19, 2022 | Vilanova de Arousa. Corón | Arousa | Bivalve | *Ruditapes philippinarum* | GYMs | TTX |
| April 19, 2022 | Vilanova de Arousa. Corón | Arousa | Gastropod | *Littorina sp.* | GYMs | TTX |
| April 19, 2022 | Vilanova de Arousa. Corón | Arousa | Cnidaria | *n.i.* | GYMs | TTX |
| April 19, 2022 | Vilanova de Arousa. Corón | Arousa | Bivalve | *Mytilus galloprovincialis* | GYMs | TTX |
| April 19, 2022 | Vilanova de Arousa. Corón | Arousa | Gastropod | *Patella sp.* | GYMs | TTX |
| April 19, 2022 | Vilanova de Arousa. Corón | Arousa | Crustacean | *Balanus sp.* | GYMs | TTX |
| April 19, 2022 | Vilanova de Arousa. Corón | Arousa | Gastropod | *Gibbula sp.* | | TTX |
| April 19, 2022 | Vilanova de Arousa. Corón | Arousa | Gastropod | *Monodonta sp.* | GYMs | TTX |
| April 19, 2022 | Vilanova de Arousa. Corón | Arousa | Gastropod | *Nassarius sp.* | GYMs | TTX |

| | | | | | | |
|---|---|---|---|---|---|---|
| April 19, 2022 | Vilanova de Arousa. Corón | Arousa | Gastropod | *Ocenebra sp.* | GYMs | TTX |
| April 19, 2022 | Vilanova de Arousa. Corón | Arousa | Gastropod | *Nucella sp.* | GYMs | TTX |
| April 19, 2022 | Carnota. Lira | Corcubión | Echinoderm | *Paracentrotus sp.* | GYMs | TTX |
| April 19, 2022 | Aldán. Vilariño | Pontevedra | Gastropod | *Crepidula sp.* | GYMs | TTX |
| April 19, 2022 | Aldán. Vilariño | Pontevedra | Gastropod | *Monodonta sp.* | GYMs | TTX |
| April 19, 2022 | Aldán. Vilariño | Pontevedra | Gastropod | *Nucella sp.* | GYMs | TTX |
| April 19, 2022 | Aldán. Vilariño | Pontevedra | Gastropod | *Gibbula sp.* | | TTX |
| April 19, 2022 | Aldán. Vilariño | Pontevedra | Gastropod | *Littorina sp.* | | TTX |
| April 19, 2022 | Aldán. Vilariño | Pontevedra | Gastropod | *Ocenebra sp.* | | TTX |
| April 19, 2022 | Aldán. Vilariño | Pontevedra | Crustacean | *Polybius sp.* | GYMs | TTX |
| April 19, 2022 | Aldán. Vilariño | Pontevedra | Cnidaria | *n.i.* | GYMs | TTX |
| April 19, 2022 | Aldán. Vilariño | Pontevedra | Cnidaria | *n.i.* | GYMs | TTX |
| April 19, 2022 | Aldán. Vilariño | Pontevedra | Gastropod | *Patella sp.* | GYMs | TTX |
| April 19, 2022 | Aldán. Vilariño | Pontevedra | Bivalve | *Mytilus galloprovincialis* | GYMs | TTX |
| April 19, 2022 | Bueu. Beluso | Pontevedra | Gastropod | *Patella sp.* | GYMs | TTX |
| April 19, 2022 | Bueu. Beluso | Pontevedra | Cnidaria | *n.i.* | GYMs | TTX |
| April 19, 2022 | Bueu. Beluso | Pontevedra | Bivalve | *Mytilus galloprovincialis* | GYMs | TTX |
| April 19, 2022 | Bueu. Beluso | Pontevedra | Gastropod | *Crepidula sp.* | GYMs | TTX |
| April 19, 2022 | Bueu. Beluso | Pontevedra | Gastropod | *Monodonta sp.* | GYMs | TTX |
| April 19, 2022 | Bueu. Beluso | Pontevedra | Gastropod | *Gibbula sp.* | | TTX |
| April 19, 2022 | Bueu. Beluso | Pontevedra | Gastropod | *Ocenebra sp.* | | TTX |
| April 19, 2022 | Barqueiro. Salgueira | Barqueiro | Bivalve | *Mytilus galloprovincialis* | GYMs | TTX |
| April 19, 2022 | Barallobre. Rampa | Ferrol | Bivalve | *Mytilus galloprovincialis* | GYMs | TTX |
| April 19, 2022 | Miño. Muro Petra Sabío | Betanzos | Bivalve | *Mytilus galloprovincialis* | GYMs | TTX |
| April 19, 2022 | Camariñas. Enseada Vasa | Camariñas | Bivalve | *Ruditapes decussatus* | GYMs | TTX |
| April 19, 2022 | Muros I | Muros-Noia | Bivalve | *Ensis ensis* | GYMs | TTX |
| April 20, 2022 | Pasaxe. Sta Cristina | A Coruña | Bivalve | *Cerastoderma edule* | GYMs | TTX |
| April 20, 2022 | Corcubión. Cee | Corcubión | Bivalve | *Mytilus galloprovincialis* | GYMs | TTX |
| April 21, 2022 | Pont V. Placeres | Pontevedra | Gastropod | *Monodonta sp.* | GYMs | TTX |
| April 21, 2022 | Pont V. Placeres | Pontevedra | Gastropod | *n.i.* | | TTX |
| April 21, 2022 | Pont V. Placeres | Pontevedra | Gastropod | *Littorina sp.* | GYMs | TTX |

| | | | | | | |
|---|---|---|---|---|---|---|
| April 21, 2022 | Pont V. Placeres | Pontevedra | Gastropod | *Patella sp.* | GYMs | TTX |
| April 21, 2022 | Pont V. Placeres | Pontevedra | Crustacean | *Polybius sp.* | GYMs | TTX |
| April 21, 2022 | Pont V. Placeres | Pontevedra | Bivalve | *Mytilus galloprovincialis* | GYMs | TTX |
| April 21, 2022 | Pont II | Pontevedra | Bivalve | *Ensis ensis* | GYMs | TTX |
| April 21, 2022 | Vigo II.2 | Vigo | Bivalve | *Ruditapes philippinarum* | GYMs | TTX |
| April 22, 2022 | Arousa I. | Arousa | Bivalve | *Venerupis rhomboides* | GYMs | TTX |
| April 22, 2022 | Pont V | Pontevedra | Bivalve | *Ruditapes philippinarum* | GYMs | TTX |
| April 26, 2022 | Barqueiro.  Salgueira | Barqueiro | Bivalve | *Mytilus galloprovincialis* | GYMs | TTX |
| April 26, 2022 | Barallobre.  Rampa | Ferrol | Bivalve | *Mytilus galloprovincialis* | GYMs | TTX |
| April 26, 2022 | Miño. Muro Petra Sabío | Betanzos | Bivalve | *Mytilus galloprovincialis* | GYMs | TTX |
| April 26, 2022 | Camariñas. Enseada Vasa | Camariñas | Bivalve | *Mytilus galloprovincialis* | GYMs | TTX |
| April 26, 2022 | Corcubión. Cee | Corcubión | Bivalve | *Mytilus galloprovincialis* | GYMs | TTX |
| April 27, 2022 | Pasaxe. Sta Cristina | A Coruña | Bivalve | *Cerastoderma edule* | GYMs | TTX |
| April 27, 2022 | Muros I | Muros-Noia | Bivalve | *Cerastoderma edule* | GYMs | TTX |
| April 28, 2022 | Pont II | Pontevedra | Bivalve | *Ensis ensis* | GYMs | TTX |
| April 28, 2022 | Pont V | Pontevedra | Bivalve | *Ruditapes philippinarum* | GYMs | TTX |
| April 29, 2022 | Arousa I. | Arousa | Bivalve | *Ruditapes philippinarum* | GYMs | TTX |
| April 29, 2022 | Barallobre.  Rampa | Ferrol | Bivalve | *Ruditapes philippinarum* | GYMs | TTX |
| May 2, 2022 | Carnota. Lira | Corcubión | Echinoderm | *Paracentrotus sp.* | GYMs | TTX |
| May 2, 2022 | Arousa. Bohido | Arousa | Bivalve | *Ruditapes philippinarum* | GYMs | TTX |
| May 2, 2022 | Carnota. Lira | Corcubión | Gastropod | *Patella depressa* | | TTX |
| May 2, 2022 | Carnota. Lira | Corcubión | Gastropod | *Patella ulyssiponensi* | GYMs | TTX |
| May 2, 2022 | Carnota. Lira | Corcubión | Gastropod | *Patella vulgata* | GYMs | TTX |
| May 2, 2022 | Carnota. Lira | Corcubión | Gastropod | *Haliotis tuberculata* | GYMs | TTX |
| May 2, 2022 | Carnota. Lira | Corcubión | Echinoderm | *Holothuria forskali* | GYMs | TTX |
| May 2, 2022 | Carnota. Lira | Corcubión | Echinoderm | *Marthasterias glacialis* | GYMs | TTX |
| May 2, 2022 | Carnota. Lira | Corcubión | Echinoderm | *Ophiothrix sp.* | | TTX |
| May 3, 2022 | Barqueiro.  Salgueira | Barqueiro | Bivalve | *Magellana gigas* | GYMs | TTX |
| May 3, 2022 | Barallobre. Maniños | Ferrol | Bivalve | *Ruditapes philippinarum* | GYMs | TTX |
| May 3, 2022 | Miño.  Lombo Espiñeira | Betanzos | Bivalve | *Ruditapes philippinarum* | GYMs | TTX |
| May 3, 2022 | Camariñas.  Enseada Vasa | Camariñas | Bivalve | *Ruditapes decussatus* | GYMs | TTX |

| | | | | | | | |
|---|---|---|---|---|---|---|---|
| May 3, 2022 | Muros I | Muros-Noia | Bivalve | *Cerastoderma edule* | GYMs | | TTX |
| May 4, 2022 | Pasaxe. Sta Cristina | A Coruña | Bivalve | *Cerastoderma edule* | GYMs | | TTX |
| May 4, 2022 | Corcubión. Cee | Corcubión | Bivalve | *Mytilus galloprovincialis* | GYMs | | TTX |
| May 4, 2022 | Pont V | Pontevedra | Bivalve | *Ensis ensis* | GYMs | | TTX |
| May 5, 2022 | Pont V | Pontevedra | Bivalve | *Ruditapes philippinarum* | GYMs | | TTX |
| May 10, 2022 | Barallobre. Maniños | Ferrol | Bivalve | *Cerastoderma edule* | GYMs | | TTX |
| May 10, 2022 | Corcubión. Cee | Corcubión | Bivalve | *Cerastoderma edule* | GYMs | | TTX |
| May 11, 2022 | Barqueiro. Salgueira | Barqueiro | Bivalve | *Magellana gigas* | GYMs | | TTX |
| May 11, 2022 | Miño. Lombo Espiñeira | Betanzos | Bivalve | *Ruditapes philippinarum* | GYMs | | TTX |
| May 11, 2022 | Camariñas. Enseada Vasa | Camariñas | Bivalve | *Ruditapes decussatus* | GYMs | | TTX |
| May 12, 2022 | Pasaxe. Sta Cristina | A Coruña | Bivalve | *Cerastoderma edule* | GYMs | | TTX |
| May 12, 2022 | Pont V. Placeres | Pontevedra | Bivalve | *Venerupis pullastra* | GYMs | | TTX |
| May 12, 2022 | Vigo II.2 Xunqueira | Vigo | Gastropod | *Haliotis sp.* | GYMs | | TTX |
| May 18, 2022 | Barqueiro. Salgueira | Barqueiro | Bivalve | *Magellana gigas* | GYMs | | TTX |
| May 18, 2022 | Barallobre. Maniños | Ferrol | Bivalve | *Cerastoderma edule* | GYMs | | TTX |
| May 18, 2022 | Miño. Lombo Espiñeira | Betanzos | Bivalve | *Ruditapes philippinarum* | GYMs | | TTX |
| May 18, 2022 | Pasaxe. Sta Cristina | A Coruña | Bivalve | *Cerastoderma edule* | GYMs | | TTX |
| May 18, 2022 | Camariñas. Enseada Vasa | Camariñas | Bivalve | *Ruditapes decussatus* | GYMs | | TTX |
| May 19, 2022 | Corcubión. Cee | Corcubión | Bivalve | *Cerastoderma edule* | GYMs | | TTX |
| May 24, 2022 | Arousa. Bohido | Arousa | Echinoderm | *Asterina gibbosa* | GYMs | 5,6,11 trideoxy TTX | TTX |
| May 24, 2022 | Arousa. Bohido | Arousa | Echinoderm | *Paracentrotus lividus* | GYMs | | TTX |
| May 24, 2022 | Arousa. Bohido | Arousa | Cnidaria | *Calliactis parasitica* | GYMs | 5,6,11 trideoxy TTX | TTX |
| May 24, 2022 | Arousa. Bohido | Arousa | Crustacean | *Nécora puber* | GYMs | 5,6,11 trideoxy TTX | TTX |
| May 24, 2022 | Arousa. Bohido | Arousa | Crustacean | *Liocarcinus corrugatus* | GYMs | 5,6,11 trideoxy TTX | TTX |
| May 24, 2022 | Arousa. Bohido | Arousa | Crustacean | *Liocarcinus arcuatus* | GYMs | 5,6,11 trideoxy TTX | TTX |
| May 24, 2022 | Arousa. Bohido | Arousa | Gastropod | *Nassarius reticulatus* | GYMs | 5,6,11 trideoxy TTX | TTX |
| May 24, 2022 | Arousa. Bohido | Arousa | Crustacean | *Atelecyclus undecimdentatus* | GYMs | 5,6,11 trideoxy TTX | TTX |
| May 24, 2022 | Miño. Lombo Espiñeira | Betanzos | Bivalve | *Ruditapes philippinarum* | GYMs | | TTX |
| May 24, 2022 | Pasaxe. Sta Cristina | A Coruña | Bivalve | *Cerastoderma edule* | GYMs | | TTX |
| May 24, 2022 | Vigo II | Vigo | Gastropod | *Haliotis sp.* | GYMs | | TTX |
| May 25, 2022 | Barqueiro. Salgueira | Barqueiro | Bivalve | *Magellana gigas* | GYMs | | TTX |

| | | | | | | | |
|---|---|---|---|---|---|---|---|
| May 25, 2022 | Camariñas. Enseada Vasa | Camariñas | Bivalve | *Ruditapes philippinarum* | GYMs | | TTX |
| May 25, 2022 | Corcubión. Cee | Corcubión | Bivalve | *Cerastoderma edule* | GYMs | | TTX |
| May 25, 2022 | Vigo I | Vigo | Bivalve | *Aequipecten opercularis* | GYMs | | TTX |
| May 26, 2022 | *Carnota. Lira* | Corcubión | Echinoderm | *Paracentrotus sp.* | GYMs | 5,6,11 trideoxy TTX | TTX |
| May 26, 2022 | Barallobre. Maniños | Ferrol | Bivalve | *Cerastoderma edule* | GYMs | | TTX |
| May 31, 2022 | Miño. Lombo Espiñeira | Betanzos | Bivalve | *Ruditapes philippinarum* | GYMs | | TTX |
| May 31, 2022 | Camariñas. Enseada Vasa | Camariñas | Bivalve | *Ruditapes philippinarum* | GYMs | | TTX |
| June 1, 2022 | Barqueiro. Salgueira | Barqueiro | Bivalve | *Magellana gigas* | GYMs | | TTX |
| June 1, 2022 | Barallobre. Maniños | Ferrol | Bivalve | *Cerastoderma edule* | GYMs | | TTX |
| June 1, 2022 | Pasaxe. Sta Cristina | A Coruña | Bivalve | *Cerastoderma edule* | GYMs | | TTX |
| June 1, 2022 | Corcubión. Cee | Corcubión | Bivalve | *Mytilus galloprovincialis* | GYMs | | TTX |
| June 7, 2022 | Miño. Lombo Espiñeira | Betanzos | Bivalve | *Ruditapes philippinarum* | GYMs | | TTX |
| June 7, 2022 | Pasaxe. Sta Cristina | A Coruña | Bivalve | *Cerastoderma edule* | GYMs | | TTX |
| June 7, 2022 | Camariñas. Enseada Vasa | Camariñas | Bivalve | *Ruditapes philippinarum* | GYMs | | TTX |
| June 7, 2022 | Corcubión. Cee | Corcubión | Bivalve | *Mytilus galloprovincialis* | GYMs | | TTX |
| June 8, 2022 | Barallobre. Rampa | Ferrol | Bivalve | *Mytilus galloprovincialis* | GYMs | | TTX |
| June 9, 2022 | Barqueiro. Salgueira | Barqueiro | Bivalve | *Mytilus galloprovincialis* | GYMs | | TTX |
| June 14, 2022 | Porto do Son. Arnela | Muros-Noia | Gastropod | *Ocenebra sp.* | | | TTX |
| June 14, 2022 | Porto do Son. Arnela | Muros-Noia | Polychaete | *n.i.* | | | TTX |
| June 14, 2022 | Porto do Son. Arnela | Muros-Noia | Polychaete | *n.i.* | GYMs | | TTX |
| June 14, 2022 | Porto do Son. Arnela | Muros-Noia | Gastropod | *Shiponaria pectinata* | GYMs | | TTX |
| June 14, 2022 | Porto do Son. Arnela | Muros-Noia | Bivalve | *Mytilus galloprovincialis* | GYMs | | TTX |
| June 14, 2022 | Porto do Son. Arnela | Muros-Noia | Crustacean | *Balanus sp.* | GYMs | | TTX |
| June 14, 2022 | Porto do Son. Arnela | Muros-Noia | Echinoderm | *Asterina sp.* | GYMs | | TTX |
| June 14, 2022 | Porto do Son. Arnela | Muros-Noia | Cnidaria | *n.i.* | GYMs | | TTX |
| June 14, 2022 | Porto do Son. Arnela | Muros-Noia | Echinoderm | *Paracentrotus sp.* | | | TTX |
| June 14, 2022 | Porto do Son. Arnela | Muros-Noia | Gastropod | *Nucella sp.* | GYMs | | TTX |
| June 14, 2022 | Porto do Son. Arnela | Muros-Noia | Gastropod | *Patella sp.* | GYMs | | TTX |
| June 14, 2022 | Porto do Son. Arnela | Muros-Noia | Gastropod | *Gibbula sp.* | | | TTX |
| June 14, 2022 | Porto do Son. Arnela | Muros-Noia | Gastropod | *Monodonta sp.* | GYMs | | TTX |
| June 14, 2022 | Vilanova de Arousa. Corón | Arousa | Cnidaria | *n.i.* | GYMs | | TTX |

| | | | | | | |
|---|---|---|---|---|---|---|
| June 14, 2022 | Vilanova de Arousa. Corón | Arousa | Gastropod | *Nassarius sp.* | GYMs | TTX |
| June 14, 2022 | Vilanova de Arousa. Corón | Arousa | Gastropod | *Monodonta sp.* | GYMs | TTX |
| June 14, 2022 | Vilanova de Arousa. Corón | Arousa | Gastropod | *Gibbula sp.* | GYMs | TTX |
| June 14, 2022 | Vilanova de Arousa. Corón | Arousa | Crustacean | *Polybius sp.* | GYMs | TTX |
| June 14, 2022 | Vilanova de Arousa. Corón | Arousa | Bivalve | *Ostrea edulis* | GYMs | TTX |
| June 14, 2022 | Vilanova de Arousa. Corón | Arousa | Crustacean | *Balanus sp.* | GYMs | TTX |
| June 14, 2022 | Vilanova de Arousa. Corón | Arousa | Gastropod | *Patella sp.* | GYMs | TTX |
| June 14, 2022 | Vilanova de Arousa. Corón | Arousa | Bivalve | *Mytilus galloprovincialis* | GYMs | TTX |
| June 14, 2022 | Vilanova de Arousa. Corón | Arousa | Gastropod | *Nucella sp.* | GYMs | TTX |
| June 14, 2022 | Vilanova de Arousa. Corón | Arousa | Gastropod | *Littorina sp.* | GYMs | TTX |
| June 14, 2022 | Vilanova de Arousa. Corón | Arousa | Gastropod | *Doris verrucosa* | GYMs | TTX |
| June 14, 2022 | Bueu. Beluso | Pontevedra | Gastropod | *Patella sp.* | GYMs | TTX |
| June 14, 2022 | Bueu. Beluso | Pontevedra | Bivalve | *Mytilus galloprovincialis* | GYMs | TTX |
| June 14, 2022 | Bueu. Beluso | Pontevedra | Gastropod | *Monodonta sp.* | GYMs | TTX |
| June 14, 2022 | Bueu. Beluso | Pontevedra | Gastropod | *Gibbula sp.* | | TTX |
| June 14, 2022 | Bueu. Beluso | Pontevedra | Gastropod | *Nucella sp.* | | TTX |
| June 14, 2022 | Bueu. Beluso | Pontevedra | Crustacean | *Balanus sp.* | | TTX |
| June 14, 2022 | Bueu. Beluso | Pontevedra | Cnidaria | *n.i.* | GYMs | TTX |
| June 14, 2022 | Aldán. Vilariño | Pontevedra | Gastropod | *Gibbula sp.* | | TTX |
| June 14, 2022 | Aldán. Vilariño | Pontevedra | Gastropod | *Nassarius sp.* | | TTX |
| June 14, 2022 | Aldán. Vilariño | Pontevedra | Gastropod | *Littorina sp.* | GYMs | TTX |
| June 14, 2022 | Aldán. Vilariño | Pontevedra | Gastropod | *Patella sp.* | GYMs | TTX |
| June 14, 2022 | Aldán. Vilariño | Pontevedra | Gastropod | *Monodonta sp.* | GYMs | TTX |
| June 14, 2022 | Aldán. Vilariño | Pontevedra | Crustacean | *Polybius sp.* | GYMs | TTX |
| June 14, 2022 | Aldán. Vilariño | Pontevedra | Cnidaria | *n.i.* | GYMs | TTX |
| June 14, 2022 | Aldán. Vilariño | Pontevedra | Bivalve | *Mytilus galloprovincialis* | GYMs | TTX |
| June 14, 2022 | Aldán. Vilariño | Pontevedra | Cnidaria | *n.i.* | GYMs | TTX |
| June 14, 2022 | Barqueiro. Salgueira | Barqueiro | Bivalve | *Mytilus galloprovincialis* | GYMs | TTX |
| June 14, 2022 | Barallobre. Rampa | Ferrol | Bivalve | *Mytilus galloprovincialis* | GYMs | TTX |
| June 14, 2022 | Miño.  Lombo Espiñeira | Betanzos | Bivalve | *Ruditapes philippinarum* | GYMs | TTX |
| June 14, 2022 | Pasaxe. Sta Cristina | A Coruña | Bivalve | *Cerastoderma edule* | GYMs | TTX |

| | | | | | | |
|---|---|---|---|---|---|---|
| June 14, 2022 | Camariñas. Enseada Vasa | Camariñas | Bivalve | *Ruditapes philippinarum* | GYMs | TTX |
| June 14, 2022 | Corcubión. Cee | Corcubión | Bivalve | *Mytilus galloprovincialis* | GYMs | TTX |
| June 14, 2022 | Camariñas. Zona E | Camariñas | Bivalve | *Ensis ensis* | GYMs | TTX |
| June 17, 2022 | Ramallosa. Esteiro Foz. | Vigo | Gastropod | *Patella sp.* | GYMs | TTX |
| June 17, 2022 | Ramallosa. Esteiro Foz. | Vigo | Bivalve | *Mytilus galloprovincialis* | GYMs | TTX |
| June 17, 2022 | Ramallosa. Esteiro Foz. | Vigo | Bivalve | *Cerastoderma edule* | GYMs | TTX |
| June 17, 2022 | Ramallosa. Esteiro Foz. | Vigo | Bivalve | *Ruditapes decussatus* | GYMs | TTX |
| June 17, 2022 | Ramallosa. Esteiro Foz. | Vigo | Gastropod | *Littorina sp.* | GYMs | TTX |
| June 17, 2022 | Ramallosa. Esteiro Foz. | Vigo | Crustacean | *Carcinus maenas* | GYMs | TTX |
| June 21, 2022 | Pont V. Placeres | Pontevedra | Gastropod | *Littorina sp.* | GYMs | TTX |
| June 21, 2022 | Pont V. Placeres | Pontevedra | Bivalve | *Mytilus galloprovincialis* | GYMs | TTX |
| June 21, 2022 | Pont V. Placeres | Pontevedra | Gastropod | *Gibbula sp.* | GYMs | TTX |
| June 21, 2022 | Pont V. Placeres | Pontevedra | Crustacean | *Polybius sp.* | GYMs | TTX |
| June 21, 2022 | Pont V. Placeres | Pontevedra | Gastropod | *Patella sp.* | GYMs | TTX |
| June 21, 2022 | Pont V. Placeres | Pontevedra | Gastropod | *Monodonta sp.* | GYMs | TTX |
| June 21, 2022 | Barallobre. Pantalán | Ferrol | Bivalve | *Mytilus galloprovincialis* | GYMs | TTX |
| June 21, 2022 | Miño. Lombo Espiñeira | Betanzos | Bivalve | *Ruditapes philippinarum* | GYMs | TTX |
| June 21, 2022 | Camariñas. Paxariñas | Camariñas | Bivalve | *Ruditapes philippinarum* | GYMs | TTX |
| June 21, 2022 | Pont II. Menduiña | Pontevedra | Bivalve | *Ensis ensis* | GYMs | TTX |
| June 21, 2022 | Camariñas. Zona E | Camariñas | Bivalve | *Ensis ensis* | GYMs | TTX |
| June 22, 2022 | Pasaxe. Sta Cristina | A Coruña | Bivalve | *Cerastoderma edule* | GYMs | TTX |
| June 23, 2022 | Barqueiro. Salgueira | Barqueiro | Bivalve | *Mytilus galloprovincialis* | GYMs | TTX |
| June 28, 2022 | Barqueiro. Salgueira | Barqueiro | Bivalve | *Mytilus galloprovincialis* | GYMs | TTX |
| June 28, 2022 | Miño. Lombo Espiñeira | Betanzos | Bivalve | *Ruditapes philippinarum* | GYMs | TTX |
| June 28, 2022 | Camariñas. Enseada Vasa | Camariñas | Bivalve | *Ruditapes philippinarum* | GYMs | TTX |
| June 29, 2022 | Barallobre. Rampa | Ferrol | Bivalve | *Mytilus galloprovincialis* | GYMs | TTX |
| June 29, 2022 | Corcubión. Cee | Corcubión | Bivalve | *Mytilus galloprovincialis* | GYMs | TTX |
| June 30, 2022 | Muros I. Cabeiro | Muros-Noia | Bivalve | *Ensis ensis* | GYMs | TTX |
| June 30, 2022 | Pont II. San Cibrao | Pontevedra | Bivalve | *Ruditapes philippinarum* | GYMs | TTX |
| June 30, 2022 | Vigo I. Subrido | Vigo | Gastropod | *Haliotis sp.* | GYMs | TTX |
| July 1, 2022 | Muros I. Pr. da Virxen | Muros-Noia | Bivalve | *Cerastoderma edule* | GYMs | TTX |

| | | | | | | | |
|---|---|---|---|---|---|---|---|
| July 5, 2022 | Barallobre. Rampa | Ferrol | Bivalve | *Mytilus galloprovincialis* | GYMs | | TTX |
| July 5, 2022 | Miño.  Lombo Espiñeira | Betanzos | Bivalve | *Ruditapes philippinarum* | GYMs | | TTX |
| July 5, 2022 | Camariñas. Enseada Vasa | Camariñas | Bivalve | *Ruditapes decussatus* | GYMs | | TTX |
| July 5, 2022 | Muros I.  Abelleira | Muros-Noia | Bivalve | *Cerastoderma edule* | GYMs | | TTX |
| July 5, 2022 | Muros I. Esteiro | Muros-Noia | Bivalve | *Ensis ensis* | GYMs | | TTX |
| July 5, 2022 | Pont II. Niño do corvo | Pontevedra | Bivalve | *Ensis ensis* | GYMs | | TTX |
| July 6, 2022 | Barqueiro. Salgueira | Barqueiro | Bivalve | *Mytilus galloprovincialis* | GYMs | | TTX |
| July 6, 2022 | Pasaxe. Sta Cristina | A Coruña | Bivalve | *Cerastoderma edule* | GYMs | | TTX |
| July 7, 2022 | Pont V | Pontevedra | Bivalve | *Ruditapes philippinarum* | GYMs | | TTX |
| July 7, 2022 | Pont II | Pontevedra | Bivalve | *Venerupis rhomboides* | GYMs | | TTX |
| July 11, 2022 | Vigo II.2 Xunqueira | Vigo | Bivalve | *Ruditapes philippinarum* | GYMs | | TTX |
| July 12, 2022 | Barqueiro. San Fiz | Barqueiro | Bivalve | *Cerastoderma edule* | GYMs | | TTX |
| July 12, 2022 | Barallobre. Rampa | Ferrol | Bivalve | *Mytilus galloprovincialis* | GYMs | | TTX |
| July 12, 2022 | Miño.  Lombo Espiñeira | Betanzos | Bivalve | *Ruditapes philippinarum* | GYMs | | TTX |
| July 12, 2022 | Camariñas. Enseada Vasa | Camariñas | Bivalve | *Ruditapes philippinarum* | GYMs | | TTX |
| July 12, 2022 | Muros I. Pr. da Virxen | Muros-Noia | Bivalve | *Cerastoderma edule* | GYMs | | TTX |
| July 12, 2022 | Pont II. Niño do corvo | Pontevedra | Bivalve | *Ensis ensis* | GYMs | | TTX |
| July 14, 2022 | Pont V. Placeres | Pontevedra | Bivalve | *Ruditapes philippinarum* | GYMs | | TTX |
| July 14, 2022 | Vigo II.2 Toralla | Vigo | Bivalve | *Venerupis pullastra* | GYMs | | TTX |
| July 14, 2022 | Vigo II.2 Con. Norte | Vigo | Bivalve | *Ruditapes philippinarum* | GYMs | | TTX |
| July 15, 2022 | Corcubión. Cee | Corcubión | Bivalve | *Cerastoderma edule* | GYMs | | |
| July 19, 2022 | Camariñas.  Enseada Vasa | Camariñas | Gastropod | *Nassarius sp.* | GYMs | | TTX |
| July 19, 2022 | Camariñas.  Enseada Vasa | Camariñas | Gastropod | *Littorina sp.* | | | TTX |
| July 19, 2022 | Camariñas.  Enseada Vasa | Camariñas | Gastropod | *Patella sp.* | GYMs | | TTX |
| July 19, 2022 | Camariñas.  Enseada Vasa | Camariñas | Bivalve | *Magellana gigas* | GYMs | | TTX |
| July 19, 2022 | Camariñas.  Enseada Vasa | Camariñas | Bivalve | *Mytilus galloprovincialis* | GYMs | | TTX |
| July 19, 2022 | Camariñas.  Enseada Vasa | Camariñas | Cnidaria | *n.i.* | GYMs | | TTX |
| July 19, 2022 | Camariñas.  Paxariñas | Camariñas | Bivalve | *Mytilus galloprovincialis* | GYMs | | TTX |
| July 19, 2022 | Camariñas.  Paxariñas | Camariñas | Crustacean | *Polybius sp.* | GYMs | | TTX |
| July 19, 2022 | Camariñas.  Paxariñas | Camariñas | Gastropod | *Littorina sp.* | GYMs | | TTX |
| July 19, 2022 | Miño.  Lombo Espiñeira | Betanzos | Bivalve | *Ruditapes philippinarum* | GYMs | | |

| | | | | | | | |
|---|---|---|---|---|---|---|---|
| July 19, 2022 | Muros I. Cabeiro | Muros-Noia | Bivalve | *Ensis ensis* | GYMs | | |
| July 19, 2022 | Pont II. Niño do Corvo | Pontevedra | Bivalve | *Ensis ensis* | GYMs | | |
| July 19, 2022 | Pont V. Placeres | Pontevedra | Bivalve | *Ruditapes philippinarum* | GYMs | | |
| July 19, 2022 | Vigo II.2. Areiño | Vigo | Bivalve | *Venerupis pullastra* | GYMs | | |
| July 20, 2022 | Barqueiro. Salgueira | Barqueiro | Bivalve | *Mytilus galloprovincialis* | GYMs | | |
| July 20, 2022 | Barallobre. Rampa | Ferrol | Bivalve | *Mytilus galloprovincialis* | GYMs | | |
| July 20, 2022 | Camariñas. Enseada Vasa | Camariñas | Bivalve | *Ruditapes philippinarum* | GYMs | | |
| July 20, 2022 | Corcubión. Cee | Corcubión | Bivalve | *Mytilus galloprovincialis* | GYMs | | |
| July 20, 2022 | Muros I. Abelleira | Muros-Noia | Bivalve | *Cerastoderma edule* | GYMs | | |
| July 21, 2022 | Pont V. Lourizán | Pontevedra | Bivalve | *Ensis ensis* | GYMs | | |
| July 21, 2022 | Pont V. Campelo | Pontevedra | Bivalve | *Ruditapes philippinarum* | GYMs | | |
| July 21, 2022 | Camariñas. Centro ría | Camariñas | Bivalve | *Ensis siliqua* | GYMs | | |
| July 26, 2022 | Vigo II.2 | Vigo | Bivalve | *Ruditapes philippinarum* | GYMs | | |
| July 27, 2022 | Barallobre. Rampa | Ferrol | Bivalve | *Mytilus galloprovincialis* | GYMs | | |
| July 27, 2022 | Camariñas. Enseada Vasa | Camariñas | Bivalve | *Ruditapes decussatus* | GYMs | | |
| July 27, 2022 | Corcubión. Cee | Corcubión | Bivalve | *Mytilus galloprovincialis* | GYMs | | |
| July 27, 2022 | Muros I. Pr.Virxen | Muros-Noia | Bivalve | *Cerastoderma edule* | GYMs | | |
| July 27, 2022 | Pont II. Niño do Corvo | Pontevedra | Bivalve | *Ensis ensis* | GYMs | | |
| July 27, 2022 | Pont V. Placeres | Pontevedra | Bivalve | *Ruditapes philippinarum* | GYMs | | |
| July 27, 2022 | Muros I. Pr.Virxen | Muros-Noia | Bivalve | *Ensis ensis* | GYMs | | |
| July 27, 2022 | Vigo II.2. Areiño | Vigo | Bivalve | *Ruditapes philippinarum* | GYMs | | |
| July 27, 2022 | Vigo II.2. A Guía | Vigo | Bivalve | *Ensis ensis* | GYMs | | |
| July 28, 2022 | Barqueiro. Salgueira | Barqueiro | Bivalve | *Mytilus galloprovincialis* | GYMs | | |
| July 28, 2022 | Miño. Lombo Espiñeira | Betanzos | Bivalve | *Ruditapes philippinarum* | GYMs | | |
| July 29, 2022 | Arousa. Bohido | Arousa | Echinoderm | *Asteria rubens* | GYMs | 5,6,11 trideoxy TTX | TTX |
| July 29, 2022 | Arousa. Bohido | Arousa | Crustacean | *Liocarcinus arcuatus* | GYMs | 5,6,11 trideoxy TTX | TTX |
| July 29, 2022 | Arousa. Bohido | Arousa | Crustacean | *Chaetopleura angulata* | GYMs | 5,6,11 trideoxy TTX | TTX |
| July 29, 2022 | Arousa. Bohido | Arousa | Crustacean | *Carcinus maenas* | GYMs | 5,6,11 trideoxy TTX | TTX |
| July 29, 2022 | Arousa. Bohido | Arousa | Crustacean | *Liocarcinus corrugatus* | GYMs | 5,6,11 trideoxy TTX | TTX |
| July 29, 2022 | Arousa. Bohido | Arousa | Gastropod | *Crepidula dilatata* | GYMs | 5,6,11 trideoxy TTX | TTX |
| August 2, 2022 | Miño. Lombo Espiñeira | Betanzos | Bivalve | *Ruditapes philippinarum* | GYMs | | |

| | | | | | | |
|---|---|---|---|---|---|---|
| August 2, 2022 | Camariñas. Enseada Vasa | Camariñas | Bivalve | *Ruditapes decussatus* | GYMs | |
| August 2, 2022 | Muros I. Abelleira | Muros-Noia | Bivalve | *Cerastoderma edule* | GYMs | |
| August 2, 2022 | Pont II. San Cibrao | Pontevedra | Bivalve | *Ruditapes philippinarum* | GYMs | |
| August 2, 2022 | Pont V. Lourizán | Pontevedra | Bivalve | *Ensis ensis* | GYMs | |
| August 2, 2022 | Vigo II.2 Domaio | Vigo | Bivalve | *Ruditapes philippinarum* | GYMs | |
| August 2, 2022 | Pont V. Placeres | Pontevedra | Bivalve | *Ruditapes philippinarum* | GYMs | |
| August 3, 2022 | Barallobre. Rampa | Ferrol | Bivalve | *Mytilus galloprovincialis* | GYMs | |
| August 3, 2022 | Corcubión. Cee | Corcubión | Bivalve | *Cerastoderma edule* | GYMs | |
| August 5, 2022 | Muros I | Muros-Noia | Bivalve | *Cerastoderma edule* | GYMs | |
| August 8, 2022 | Muros I. Abelleira | Muros-Noia | Bivalve | *Cerastoderma edule* | GYMs | |
| August 9, 2022 | Corcubión. Cee | Corcubión | Bivalve | *Mytilus galloprovincialis* | GYMs | |
| August 9, 2022 | Pont V. Placeres | Pontevedra | Bivalve | *Ruditapes philippinarum* | GYMs | |
| August 9, 2022 | Vigo II.2 Xunqueira | Vigo | Bivalve | *Ruditapes philippinarum* | GYMs | |
| August 10, 2022 | Barqueiro. Salgueira | Barqueiro | Bivalve | *Mytilus galloprovincialis* | GYMs | |
| August 10, 2022 | Miño. Lombo Espiñeira | Betanzos | Bivalve | *Ruditapes philippinarum* | GYMs | |
| August 10, 2022 | Camariñas. Paxariñas | Camariñas | Bivalve | *Ruditapes decussatus* | GYMs | |
| August 17, 2022 | Miño. Lombo Espiñeira | Betanzos | Bivalve | *Ruditapes philippinarum* | GYMs | |
| August 17, 2022 | Muros I. Abelleira | Muros-Noia | Bivalve | *Cerastoderma edule* | GYMs | |
| August 18, 2022 | Barqueiro. Salgueira | Barqueiro | Bivalve | *Mytilus galloprovincialis* | GYMs | |
| August 18, 2022 | Barallobre. Pantalán | Ferrol | Bivalve | *Mytilus galloprovincialis* | GYMs | |
| August 18, 2022 | Camariñas. Paxariñas | Camariñas | Bivalve | *Ruditapes philippinarum* | GYMs | |
| August 18, 2022 | Corcubión. Cee | Corcubión | Bivalve | *Cerastoderma edule* | GYMs | |
| August 18, 2022 | Arousa I. Moreiras | Arousa | Bivalve | *Ruditapes philippinarum* | GYMs | |
| August 18, 2022 | Vigo II.2. Areiño | Vigo | Bivalve | *Venerupis pullastra* | GYMs | |
| August 19, 2022 | Pont V. Placeres | Pontevedra | Bivalve | *Ruditapes philippinarum* | GYMs | |
| September 10, 2022 | Nerga. Os Castros | Vigo | Echinoderm | *Paracentrotus sp.* | GYMs | TTX |
| September 10, 2022 | Nerga. Os Castros | Vigo | Gastropod | *Monodonta sp.* | GYMs | TTX |
| September 10, 2022 | Nerga. Os Castros | Vigo | Gastropod | *Patella sp.* | GYMs | TTX |
| September 10, 2022 | Nerga. Os Castros | Vigo | Cnidaria | *n.i.* | GYMs | TTX |
| September 10, 2022 | Nerga. Os Castros | Vigo | Crustacean | *n.i.* | GYMs | TTX |
| September 26, 2022 | Vilanova de Arousa. Corón | Arousa | Bivalve | *Mytilus galloprovincialis* | GYMs | TTX |

| | | | | | | | |
|---|---|---|---|---|---|---|---|
| September 26, 2022 | Vilanova de Arousa. Corón | Arousa | Gastropod | *Gibbula sp.* | | | TTX |
| September 26, 2022 | Vilanova de Arousa. Corón | Arousa | Crustacean | *n.i.* | GYMs | 5,6,11 trideoxy TTX | TTX |
| September 26, 2022 | Vilanova de Arousa. Corón | Arousa | Gastropod | *Littorina sp.* | GYMs | | TTX |
| September 26, 2022 | Vilanova de Arousa. Corón | Arousa | Gastropod | *Monodonta sp.* | GYMs | | TTX |
| September 26, 2022 | Vilanova de Arousa. Corón | Arousa | Gastropod | *Nucella sp.* | GYMs | | TTX |
| September 26, 2022 | Vilanova de Arousa. Corón | Arousa | Gastropod | *Doris verrucosa* | GYMs | | TTX |
| September 26, 2022 | Vilanova de Arousa. Corón | Arousa | Gastropod | *Patella sp.* | GYMs | | TTX |
| September 26, 2022 | Vilanova de Arousa. Corón | Arousa | Cnidaria | *n.i.* | GYMs | | TTX |
| September 26, 2022 | Vilanova de Arousa. Corón | Arousa | Gastropod | *Ocenebra sp.* | | | TTX |
| September 27, 2022 | Bueu. Cabo Udra | Pontevedra | Gastropod | *Gibbula magus* | | | TTX |
| September 27, 2022 | Bueu. Cabo Udra | Pontevedra | Gastropod | *Patella sp.* | GYMs | | TTX |
| September 27, 2022 | Porto do Son. Arnela | Muros-Noia | Gastropod | *Patella sp.* | GYMs | | TTX |
| September 27, 2022 | Porto do Son. Arnela | Muros-Noia | Echinoderm | *Asterina sp.* | GYMs | | TTX |
| September 27, 2022 | Porto do Son. Arnela | Muros-Noia | Gastropod | *Nassarius sp.* | | | TTX |
| September 27, 2022 | Porto do Son. Arnela | Muros-Noia | Polychaete | *n.i.* | GYMs | | TTX |
| September 27, 2022 | Porto do Son. Arnela | Muros-Noia | Gastropod | *Patella sp.* | GYMs | | TTX |
| September 27, 2022 | Porto do Son. Arnela | Muros-Noia | Bivalve | *Mytilus galloprovincialis* | GYMs | | TTX |
| September 27, 2022 | Porto do Son. Arnela | Muros-Noia | Cnidaria | *n.i.* | GYMs | | TTX |
| September 27, 2022 | Porto do Son. Arnela | Muros-Noia | Crustacean | *n.i.* | GYMs | | TTX |
| September 27, 2022 | Porto do Son. Arnela | Muros-Noia | Gastropod | *Aplysia punctata* | GYMs | | TTX |
| September 27, 2022 | Porto do Son. Arnela | Muros-Noia | Gastropod | *Monodonta sp.* | GYMs | | TTX |
| September 27, 2022 | Porto do Son. Arnela | Muros-Noia | Gastropod | *Gibbula sp.* | GYMs | | TTX |
| September 27, 2022 | Porto do Son. Arnela | Muros-Noia | Gastropod | *Nucella sp.* | GYMs | | TTX |
| September 28, 2022 | Bueu. Beluso | Pontevedra | Cnidaria | *n.i.* | GYMs | | TTX |
| September 28, 2022 | Bueu. Beluso | Pontevedra | Bivalve | *Mytilus galloprovincialis* | GYMs | | TTX |
| September 28, 2022 | Bueu. Beluso | Pontevedra | Echinoderm | *Paracentrotus sp.* | GYMs | | TTX |
| September 28, 2022 | Bueu. Beluso | Pontevedra | Gastropod | *Crepidula sp.* | GYMs | | TTX |
| September 28, 2022 | Bueu. Beluso | Pontevedra | Crustacean | *n.i.* | GYMs | | TTX |
| September 28, 2022 | Bueu. Beluso | Pontevedra | Bivalve | *Ruditapes philippinarum* | GYMs | | TTX |
| September 28, 2022 | Bueu. Beluso | Pontevedra | Gastropod | *Nucella sp.* | GYMs | | TTX |
| September 28, 2022 | Bueu. Beluso | Pontevedra | Gastropod | *Gibbula sp.* | | | TTX |

| | | | | | | |
|---|---|---|---|---|---|---|
| September 28, 2022 | Bueu. Beluso | Pontevedra | Gastropod | *Monodonta sp.* | GYMs | TTX |
| September 28, 2022 | Bueu. Beluso | Pontevedra | Gastropod | *Patella sp.* | GYMs | TTX |
| September 28, 2022 | Aldán. Vilariño | Pontevedra | Bivalve | *Mytilus galloprovincialis* | GYMs | TTX |
| September 28, 2022 | Aldán. Vilariño | Pontevedra | Bivalve | *Ostrea edulis* | GYMs | TTX |
| September 28, 2022 | Aldán. Vilariño | Pontevedra | Bivalve | *Ruditapes philippinarum* | GYMs | TTX |
| September 28, 2022 | Aldán. Vilariño | Pontevedra | Gastropod | *Patella sp.* | GYMs | TTX |
| September 28, 2022 | Aldán. Vilariño | Pontevedra | Crustacean | *n.i.* | GYMs | TTX |
| September 28, 2022 | Aldán. Vilariño | Pontevedra | Gastropod | *Monodonta sp.* | GYMs | |
| September 28, 2022 | Aldán. Vilariño | Pontevedra | Sea squirt | *n.i.* | GYMs | |
| September 28, 2022 | Aldán. Vilariño | Pontevedra | Gastropod | *Crepidula sp.* | GYMs | |
| September 28, 2022 | Aldán. Vilariño | Pontevedra | Gastropod | *Nucella sp.* | GYMs | |
| September 28, 2022 | Aldán. Vilariño | Pontevedra | Sea squirt | *Fhallusia manillata* | GYMs | |
| September 28, 2022 | Muros. Tal | Muros-Noia | Gastropod | *Littorina sp.* | GYMs | |
| September 28, 2022 | Muros. Tal | Muros-Noia | Crustacean | *Balanus sp.* | | |
| September 29, 2022 | O Grove. Pedras negras | Arousa | Cnidaria | *n.i.* | GYMs | |
| October 5, 2022 | Aldán. Vilariño | Pontevedra | Sea squirt | *Ascidiiae indet* | GYMs | |
| October 11, 2022 | Arousa. Bohido | Arousa | Crustacean | *Liocarcinus corrugatus* | GYMs | |
| October 11, 2022 | Arousa. Bohido | Arousa | Crustacean | *Carcinus maenas* | GYMs | |
| October 11, 2022 | Arousa. Bohido | Arousa | Crustacean | *Liocarcinus arcuatus* | GYMs | |
| October 17, 2022 | Rianxo. Porrón | Arousa | Cnidaria | *n.i.* | GYMs | |

*n.i.* Unidentified
